# Atopic dermatitis: diagnosis, molecular pathogenesis, and therapeutics

**DOI:** 10.1186/s43556-025-00313-3

**Published:** 2025-10-06

**Authors:** Ruimin Bai, Yan Zheng, Xiaofeng Dai

**Affiliations:** 1 Department of Dermatology, The First Affiliated Hospital of Xi’an Jiaotong University, Xi’an Jiaotong University, No.277 Yanta West Road, Xi’an, Shaanxi 710061 China; 2 National Local Joint Engineering Research Center for Precision Surgery & Regenerative Medicine, Shaanxi Provincial Center for Regenerative Medicine and Surgical Engineering, The First Affiliated Hospital of Xi’an Jiaotong University, Xi’an Jiaotong University, No.277 Yanta West Road, Xi’an, Shaanxi 710061 China

**Keywords:** Atopic dermatitis, Diagnosis, Pathogenesis, Therapeutics, Wound healing model, Cold atmospheric plasma

## Abstract

Atopic dermatitis (AD) is a chronic inflammatory skin disease characterized by acute and chronic phases with no definitive cure currently available. The diagnosis of AD involves the evaluation of both disease onset and severity, relying on established clinical criteria and, increasingly, on various biomarkers to improve diagnostic accuracy. The molecular pathogenesis of AD is driven by a combination of genetic predispositions, environmental factors, and immune dysregulation. Acute AD is predominantly mediated by T-helper cell 2 (Th2) immune responses, whereas chronic AD involves a shift toward Th1-driven inflammation. Within this immunological context, we emphasize the role of redox imbalance in disease progression and propose a wound-healing model to explain the molecular dynamics of AD. According to this model, the acute phase is marked by excessive oxidative stress, requiring antioxidant intervention, whereas the chronic phase is characterized by insufficient redox signaling, which hinders the clearance of hyperproliferative cells. We further review current and emerging therapeutic strategies, including anti- and pro-oxidative strategies, based on the different AD staging. Notably, we introduced cold atmospheric plasma (CAP), a redox regulatory tool, as a novel treatment modality for AD management that stimulates antioxidant responses at low to moderate doses and induces oxidative stress at higher concentrations, potentially reversing chronic AD pathology. This review offers a comprehensive overview of AD, from clinical manifestations and molecular pathogenesis to therapeutic approaches, and introduces the ‘wound healing model’ as a conceptual framework to integrate CAP as an innovative treatment modality for AD management and to inform future research.

## Introduction

Atopic dermatitis (AD), first identified in the 1930 s, is the most common inflammatory dermatosis. It manifests as chronic, recurrent eczema-like lesions and intense itching, which imposes a heavy psychological burden on patients. The word ‘atopic’ originated from the Greek word ‘atopos,’ meaning that the symptom is a widespread systemic allergic response [1, 2]. AD is currently incurable and has an increased risk of comorbidities with many diseases, such as asthma, allergic rhinitis, psychiatric disorders, and skin cancer [1, 3, 4]. As one of the most burdensome skin conditions, ranking as the 15th most common nonfatal disease globally [2, 3, 5, 6], AD afflicts approximately 15%–20% of children and 10% of adults. Approximately 80% of AD cases appear in childhood, with the prevalence peaking at ages 5–9 years and declining throughout adulthood. The rest of approximately 20% of patients with AD remain asymptomatic until adulthood [2, 3, 7]. While some patients may experience gradual resolution of skin symptoms, individuals with later disease onset or more severe symptoms may have a higher risk of disease persistence [8]. Trends in AD from 1990 to 2021 were evaluated by analyzing data from the Global Burden of Disease Study [9]. The findings revealed that the total number of AD cases worldwide increased from 107.5 million (95% Uncertainty Interval (UI): 103.0–112.1 million) in 1990 to 129 million (95% UI: 123.9–134.2 million) in 2021 [9]. Despite this overall rise, the age-standardized prevalence declined from 1,885.4 per 100,000 individuals (95% UI: 1,809.0–1,962.3) in 1990 to 1,728.5 (95% UI: 1,658.5–1,798.6) in 2021 [9]. Similarly, the number of incident AD cases increased by 18.5%, from 13.5 million (95% UI: 12.8–14.2 million) in 1990 to 16 million (95% UI: 15.2–16.8 million) in 2021 [9]. However, the age-standardized incidence rate showed a slight decline over the same period, decreasing from 234.8 per 100,000 individuals (95% UI: 223.0–247.1) in 1990 to 220.6 (95% UI: 209.5–232.0) in 2021 [9]. The International Study of Asthma and Allergies in Childhood (ISAAC) directly compared data from approximately 2 million children across 100 countries worldwide using a standardized approach and showed that the incidence of AD was higher in females than in Males, and the prevalence of AD has been continuously growing globally, particularly in low-income regions and among children aged 6–7 years and 13–14 years [6, 10].

The pathogenesis of AD begins with damage to the skin barrier, which allows antigens to penetrate the skin. The skin barrier primarily refers to the intact structure of the stratum corneum, which is composed of corneocytes, structural proteins, and lipids and is formed by epidermal keratinocyte terminal differentiation [11]. In AD, the stratum corneum architecture is disrupted, leading to a “leaky” barrier that is more susceptible to external irritants [11]. It is worth noting that barrier dysfunction may not only occur in lesional skin but also in seemingly healthy non-lesional skin in patients with AD [11]. Once antigens penetrate the compromised skin barrier, they activate antigen-presenting cells (APCs), which subsequently trigger the activation of T helper cell 2 (Th2), further promoting the production of a number of cytokines. These cytokines, in turn, lead to class switching of immunoglobulin E (IgE) and the activation of eosinophils and mast cells, leading to the development of itchiness and other acute symptoms that later progress to the chronic phase if left untreated [12]. This mechanism is known as the "outside-in" hypothesis [8]. An alternative theory, referred to as the "inside-out" hypothesis, proposes that immune dysfunction in AD occurs first, triggering skin inflammation, which subsequently leads to barrier impairment and microbial dysbiosis, thereby exacerbating allergen penetration [8].

Current treatment strategies for AD include topical and systemic therapies. Traditional topical therapies include glucocorticoids, calcineurin inhibitors, and ultraviolet (UV) phototherapy. Novel topical therapies include transient receptor potential vanilloid 1 (TRPV1) antagonist, Janus kinase (JAK) inhibitors, aryl hydrocarbon receptor (AhR) agonists, and phosphodiesterase 4 (PDE4) inhibitors. Systemic therapies, oral immunosuppressants, and biologics, such as intravenous IL-targeting monoclonal antibodies: dupilumab (targeting IL-4 receptor alpha [IL-4Rα]), tralokinumab and lebrikizumab (targeting IL-13), and nemolizumab (targeting IL-31). Systemic oral formulations of JAK and PDE4 inhibitors have also been developed [1, 13]. However, all of these aforementioned therapeutic approaches have unavoidable side effects [1, 2, 7, 14–16]. Therefore, it is of paramount importance to investigate innovative treatment modalities for effective AD management with minimal adverse effects, for which an in-depth understanding of AD pathogenesis is a prerequisite.

Therefore, this study proposes a novel pathogenesis model of AD, namely the ‘Wound Healing Model,’ from the redox modulation point of view. In addition, this model states that the pathogenesis of AD is characterized by an imbalance in redox homeostasis that differs between the acute and chronic phases. That is, while the acute phase is characterized by overt oxidation, the chronic phase is characterized by reduction. Accordingly, redox modulatory tools, such as cold atmospheric plasma (CAP), may be desirable for treating acute AD by stimulating cells’ anti-oxidant machinery and resolving chronic AD by reversing cells back to the acute phase or pushing them to programmed death. To achieve this, this study introduces the canonical views on AD diagnosis, manifestations, and pathogenesis as background information. We principally underpin the criticality of redox homeostasis in acute and chronic AD, bring forth the ‘wound healing model’ to decode the implication of redox balance in treating AD of different phases, categorize current therapeutics according to their roles in redox perturbation, and propose the unique benefits of CAP in managing AD.

## Diagnosis and manifestations of atopic dermatitis

### Diagnosis of atopic dermatitis

The increasing global prevalence of AD has amplified the critical role of precise diagnostic methods. Given the heterogeneous presentation of AD, overlapping features with conditions such as psoriasis, seborrheic dermatitis, and contact dermatitis, an accurate diagnosis of AD onset and severity is paramount to avoid mismanagement.

#### Diagnosis of atopic dermatitis onset

The earliest and most widely recognized criterion for diagnosing AD was established by Hanifin and Rajka in 1980 [17] (Table 1). This standard requires that patients meet at least three of the four Major criteria and three of the 23 minor criteria [17]. The four major criteria include itching (pruritus), typical morphology and distribution of skin lesions (lichenification or flexural patterns in adults, and facial and extensor involvement in children), chronic or relapsing course of dermatitis, and personal or family history of atopic diseases such as asthma, allergic rhinitis, or AD. The 23 minor criteria provide a detailed description of clinical AD features, including but not limited to xerosis (dry skin), ichthyosis/palmar hyperlinearity/keratosis pilaris, immediate (type I) skin reactivity, elevated serum IgE, early age of onset, increased susceptibility to skin infections (especially ‘Staphylococcus aureus' and herpes simplex), hand and foot dermatitis, nipple eczema, cheilitis (lip inflammation), recurrent conjunctivitis, Dennie-Morgan folds, keratoconus, anterior subcapsular cataracts, periorbital darkening, pale or erythematous facial skin, pityriasis alba, anterior neck folds, pruritus with sweating, sensitivity to wool, perifollicular accentuation, food intolerance, disease influenced by environmental or emotional factors, white dermatographism/delayed blanching [17].

**Table 1:** Summary of diagnostic criteria for AD

Criteria	Target Population	Major Criteria	Minor Criteria/Additional Features	Diagnostic Threshold	Reference
Hanifin & Rajka (1980)	General	• Itching • Typical morphology •Chronic/relapsing course • Personal/family history of atopy	• Xerosis (dry skin) • Ichthyosis/palmar hyperlinearity/keratosis pilaris, • Immediate (type I) skin reactivity • Elevated serum IgE • Early age of onset • Increased susceptibility to skin infections (especially ‘Staphylococcus aureus' and herpes simplex) • Hand and foot dermatitis • Nipple eczema • Cheilitis (lip inflammation) • Recurrent conjunctivitis • Dennie-Morgan folds • Keratoconus • Anterior subcapsular cataracts • Periorbital darkening • Pale or erythematous facial skin • Pityriasis alba • Anterior neck folds • Pruritus with sweating • Sensitivity to wool • Perifollicular accentuation • Food intolerance • Disease influenced by environmental or emotional factors • White dermatographism/delayed blanching	At least 3 major + at least 3 minor	[17]
Williams Criteria (1993) [18]	General (esp. pediatric use)	• Itchy skin condition (or parental report)	• Skin creases involvement • Personal/family history of atopy • Dry skin in last year • Visible flexural eczema • Onset before age 2	Must meet the major criterion + at least 3 minor	[18]
Zhang Criteria (2016) [19]	Adolescents & Adults	• Symmetrical eczema > 6 months	• Personal/family history of atopy • Elevated total IgE and/or eosinophilia and/or positive specific IgE	1 major + at least 1 minor	[19]
Yao Criteria (2019) [20, 21]	Infants (0–1 year) [21]	• Rash onset after 2 weeks • Pruritus and/or irritability/sleep disturbance	• Eczema on cheeks, scalp, or extensors • Eczema elsewhere with xerosis	2 major + at least 1 minor	[21]
	Children (1–12 years) [20]	• Pruritus •Typical/atypical rash with xerosis • Chronic or relapsing course		All 3 required	[20]

*Abbreviation*: *IgE* immunoglobulin E

In addition to the widely recognized Hanifin and Rajka criteria, the Williams criteria were established in 1993 to provide a more streamlined diagnostic approach for AD [18] (Table 1). The Williams criteria require the presence of an itchy skin condition (or parental report of scratching or rubbing in a child) as a mandatory feature, along with at least three of the following minor criteria: (1) a history of involvement in skin creases (such as the folds of the elbows, knees, ankles, neck, or around the eyes); (2) a personal history of asthma or hay fever, or atopic disease in a first-degree relative (for children under 4 years old); (3) a history of generally dry skin in the past year; (4) visible flexural eczema (or involvement of the cheeks, forehead, and extensor Limbs in children under 4 years old); and (5) disease onset before age 2 (this criterion is not applied to children under 4 years old). The original 1994 version also highlighted cheek involvement in young children [18]. In 2016, the Zhang criteria were developed based on the clinical experience in China and were particularly tailored for adolescents and adults (Table 1) [19]. These criteria included one Major criterion, symmetrical eczema persisting for more than 6months, and two minor criteria: (1) a personal and/or family history of atopic diseases and (2) elevated total serum IgE or peripheral eosinophilia and/or at least one positive specific IgE test. A diagnosis can be Made if one Major criterion and at least one minor criterion are present. For infants and children, the Yao criteria were proposed in 2019 (Table 1) [20, 21]. Specifically, for infants aged 0–1 year, the diagnostic criteria included (1) the onset of rash after two weeks of age and (2) associated pruritus and/or irritability or sleep disturbances. Additionally, either of the following features must be present: (1) eczematous lesions on the cheeks, scalp, or extensor limbs or (2) eczematous lesions on other body areas accompanied by dry skin. Diagnosis requires both essential criteria and at least one of the two listed skin Manifestations. In children aged 1–12 years, the diagnosis is based on three features: (1) itching, (2) typical flexural eczema or atypical eczema with xerosis, and (3) chronic or chronically relapsing course.

The American Academy of Dermatology has developed its criteria for diagnosing AD, with key diagnostic features being chronic or relapsing eczema and pruritus. Important characteristics include onset in infancy (typically between 2–6 months of age), personal or family history of allergic diseases, elevated total serum IgE, dry skin, and additional signs, including the presence of Hertoghe’s sign (thinning of the lateral eyebrows) and hyperlinear palms [2]. For atypical AD cases, a skin biopsy may be necessary to rule out other similar skin conditions, such as seborrheic dermatitis, psoriasis, allergic or irritant contact dermatitis, and lichen planus-like keratosis. Such biopsies include the detection of primary ichthyoses, scabies, fungal infections, HIV-related skin conditions, and malignancies such as cutaneous T-cell lymphoma [18].

Emerging molecular technologies have revolutionized the diagnosis of AD by leveraging non-invasive sampling, including tape strips and swabs, minimally invasive blood tests, and lesional biopsies to profile biomarkers across multi-omics layers, including transcriptomic, proteomic, lipidomic, and microbiome analyses [22, 23]. High-throughput sequencing and computational pipelines have identified mitochondrial dysfunction as a novel axis in priming AD pathogenesis using a curated panel of mitochondrial-related differentially expressed genes (MitoDEGs) derived from the MitoCarta 3.0 and GEO datasets. This enabled early diagnosis of AD onset by interrogating dysregulated pathways in oxidative phosphorylation (OXPHOS) and apoptosis using an integrated molecular panel comprised of bcl-2-associated X protein (BAX), isocitrate dehydrogenase 3 [NAD] subunit alpha (IDH3A), mitochondrial ribosomal protein S 6 (MRPS6), and glutamic-pyruvic transaminase 2 (GPT2) [24]. Additionally, upregulated levels of Th2-skewed mediators, including IL-13, chemokine (C–C motif) Ligand 17 (CCL17), IgE, and TSLP, and induced expression of the barrier dysfunction indicator transepidermal water loss (TEWL), together with downregulated natural moisturizing factor (NMF) levels, have been used to predict AD flare-ups [23]. Some microRNAs, notably miR-155 and miR-146a, have been identified as potential diagnostic biomarkers for AD onset [24, 25]. These advances bridge bench-to-bedside translation, enabling precision-onset diagnostics that decode AD heterogeneity while guiding targeted interventions (Table 2).

**Table 2:** Key biomarkers for diagnosis of AD

Diagnostic Implication	Biomarkers	Category	Reference
Diagnosis of AD onset	BAX, IDH3A, MRPS6, GPT2	Mitochondrial-related genes	[19]
Diagnosis of AD onset	IL-13, CCL17, TSLP	Th2-skewed cytokines/chemokines	[18]
Diagnosis of AD onset	IgE	Immunoglobulin	[18]
Diagnosis of AD onset	TEWL, NMF	Skin barrier markers	[18]
Diagnosis of AD onset	miR-155, miR-146a	MicroRNAs	[19, 20]
Diagnosis of AD severity	CCL17, CCL18, CCL22, CCL26, CCL27	Chemokines	[21]
Diagnosis of AD severity	IL-13, IL-16, IL-18, IL-19, IL-22, IL-31, IL-33	Cytokines	[18, 21]
Diagnosis of AD severity	Cluster 1 (↑PARC, TIMP-1, CD14); Cluster 2 (↓IFN-α, TIMP1, VEGF); Cluster 3 (↓IFN-β, IL-1); Cluster 4 (↑IL-1/IL-4/IL-13/TSLP)	Inflammatory clusters (based on serum mediators)	[23]
Diagnosis of AD severity	S100A7, S100A8, S100A9, S100A12	AMPs	[18, 21]
Diagnosis of AD severity	ECP, EDN	Eosinophil-related	[17]
Diagnosis of AD severity	IgE	Allergen-specific markers	[23]
Diagnosis of AD severity	PGF2α, PGE2, PGD2	Prostaglandins	[18]
Diagnosis of AD severity	FLG, Loricrin, NMF	Skin barrier proteins	[18, 21]

*Abbreviations*: *BAX* bcl-2-associated X protein, *IDH3A* isocitrate dehydrogenase 3 [NAD] subunit alpha, *MRPS6* mitochondrial ribosomal protein S6, *GPT2* glutamic-pyruvic transaminase 2, *IL* interleukin, *CCL* chemokine (C–C motif) ligand, *TSLP* thymic stromal lymphopoietin, *IgE* immunoglobulin E, *TEWL* trans-epidermal water loss, *NMF* natural moisturizing factor, *PARC* p53-associated parkin-like cytoplasmic protein, *TIMP* tissue inhibitor of metalloproteinases, *IFN* interferon, *VEGF* vascular endothelial growth factor, *AMPs* antimicrobial peptides, *ECP* eosinophil cationic protein, *EDN* eosinophil-derived neurotoxin, *PGF2α* prostaglandin F2 alpha, *PGE2* prostaglandin E2, *PGD2* prostaglandin D2, *FLG* filaggrin

#### Diagnosis of atopic dermatitis severity

The severity of AD can be quantified using several scoring systems, including the eczema area and severity index (EASI) and the SCORing AD (SCORAD) scale. The EASI scale evaluates the severity of skin lesions based on erythema, edema/infiltration/papulation, scaling, and lichenification, and assesses the percentage of body area affected across four regions: head, neck, upper Limbs, trunk, and lower Limbs. The scores for each area were combined to yield a total score ranging from 0 to 72. Scores of 7 or below indicate mild AD, 8–21 indicate moderate, 22–50 indicate severe, and 51–72 indicate very severe AD [2]. In contrast, the SCORAD scale includes a broader range of lesion types and affected areas. It assesses the extent of skin involvement across areas such as the head and neck, upper limbs, lower limbs, chest, back, and groin, and evaluates lesion severity based on features such as erythema/discoloration, edema/papules, oozing/crusting, erosion, lichenification/prurigo, and dryness. Additionally, the SCORAD incorporates patient-reported symptom severity using two visual analog scales to quantify the degree of itching and sleep loss experienced over the past 3 days (including nighttime). SCORAD scores range from 0 to 103, with scores of 25 or below indicating mild AD, 26–50 being associated with moderate AD, and 51–103 indicative of severe AD [2].

The role of biomarkers in the characterization of AD severity has been previously recognized. Several candidate biomarkers have been proposed to be associated with AD severity, including CCL17/18/22/26/27 and IL13/16/18/19/22/31/33 [26]. It has been reported that chemokines and interleukins were up-regulated in AD and accelerated the disease progression via contributing to inflammation and immune dysregulation [23, 26]. Based on 131 serum biomarkers, patients with AD were classified into high- and low-inflammation groups, with the high-inflammation group exhibiting a broader inflammation marker profile associated with higher disease severity [22, 27]. Furthermore, patients with moderate-to-severe AD can be stratified into eosinophil-high and eosinophil-low subtypes, with the high-expression subtype associated with more severe transcriptional dysregulation [22]. In another study, by analyzing 147 serum mediators together with total and 130 allergen-specific IgE levels, patients with AD were categorized into four distinct groups, i.e., cluster 1 as characterized by elevated levels of pulmonary and activation-regulated chemokine (PARC), tissue inhibitor of metalloproteinases 1 (TIMP1), and CD14, cluster 2 as featured by reduced levels of interferon-α (IFN-α), TIMP1, and vascular endothelial growth factor (VEGF), cluster 3 as characterized by the lowest levels of IFN-β, IL-1, and epithelial-derived cytokines, and cluster 4 as featured by heightened levels of inflammatory markers IL-1/IL-4/IL-13 and TSLP. These clusters have been associated with distinct severities based on SCORAD (i.e., scoring atopic dermatitis severity), with clusters 4 and 2 being associated with the highest and lowest severities, respectively, and clusters 1 and 3 with intermediate severities [28]. In addition, emerging translational insights into immune-inflammatory pathways are unveiling novel biomarker candidates for stratifying AD severity, with antimicrobial peptides (AMPs) from the S100 calcium-binding protein family (S100A7/A8/A9/A12), eosinophil-derived mediators such as eosinophil cationic protein (ECP) and eosinophil-derived neurotoxin (EDN), allergen-specific IgE, and pro-inflammatory prostaglandins (i.e., prostaglandin F2 alpha (PGF2α), prostaglandin E2 (PGE2), prostaglandin D2 (PGD2)) demonstrating potential. Specifically, S100 calcium-binding family proteins reflect keratinocyte stress and microbial susceptibility, elevated ECP/EDN levels mirror eosinophil-driven tissue damage, IgE titers indicate Th2-polarized sensitization, and prostaglandin profiles map dynamic shifts between the acute inflammation (PGE2-dominated) and chronic remodeling (PGD2-predominant) phases. In contrast, down-regulated levels of skin barrier-associated proteins such as filaggrin (FLG), loricrin, and NMF have been associated with impaired epidermal function and advanced AD severity [23, 26]. Collective quantification of these markers may provide additional insights into AD endotypes, the severity of precise therapeutics, and dynamic monitoring. Table 2 shows the key biomarkers of AD.

### Manifestations of atopic dermatitis

#### Manifestations of acute and chronic atopic dermatitis

Clinically, AD has two stages: acute and chronic. Basic clinical features of AD include pruritus and eczema. Acute lesions are characterized by diffuse erythema and exuding papules [29, 30]. The pathological manifestations of acute AD include spongiosis and perivascular dermal infiltration of T cells and macrophages [31]. Chronic AD lesions are poorly demarcated, with scaly plaques accompanied by epidermal detachment and mossy lesions, and chronic AD is associated with stronger cell proliferation and increased expression of keratin 16 and Ki67 [29, 30]. The pathological manifestations of chronic AD include hyperkeratosis, acanthosis, and perivascular dermal infiltration by T cells, macrophages and mastocytes [31]. While the acute phase of AD is predominantly mediated by the Th2 immune reaction, the chronic phase is characterized by an activated Th1 immune response that involves Th2, Th17, and Th22 cells. It is believed that Th2 cells are activated in acute AD, and more Th1 cells expressing IFN-γ are involved in the chronic phase of AD. For example, an atopic patch test induced by house dust mite antigens demonstrated a transition from Th2 to Th1 in the pathogenesis of chronic AD [30]. Therefore, AD is considered a bidirectional T cell-mediated disease, with Th2 signaling predominating in the acute phase and a Th2-to-Th1 transition featuring the commencement of chronic AD [30].

It is also considered that the progression of acute AD to the chronic stage is accompanied by increased expression of Th1 (e.g., IFN-γ, CXCL9, CXCL10), Th2 (e.g., IL-4, IL-13), and Th22 markers (IL-22); and IL-4/IL-13 levels are higher than IFN-γ in both patients with acute and chronic AD [32, 33]. In addition, significant upregulation of Th2-related genes including IL-4, IL-13, IL-10, IL-31, CCL11, Th22-related genes such as IL-22, S100A7, S100A8, S100A9, S100A12, and IL-32 were detected in the lesions of patients with chronic AD, and increased Th2/Th22 signaling was intertwined with the upregulation of Th1-related genes (e.g., IFN-γ, CXCL9, CXCL10 and CXCL11) in the lesions of patients with chronic AD [30]. In addition, Th17 polarization is significantly increased in some patients with chronic AD [34]. It has been suggested that Th2-type cytokines, such as IL-4/IL-13, inhibit the polarization of the immune system from Th2 to Th1 and Th17 responses [35, 36]. For example, IL-17A inhibits the expression of TSLP and Th2 cytokines, and IL-4 inhibits the function of IL-17A, suggesting negative reciprocal regulation of the Th17 and Th2 pathways in a human skin model [33]. As chronic AD mice have more severe disease manifestations [37], it is possible that both Th2 and Th17 responses are increased in the chronic phase of AD in addition to Th1. The possible existence of mutual antagonism among these pathways controls disease progression and contributes to the severe and complicated pathological manifestations of chronic AD that render its management challenging.

#### Manifestations of extrinsic and intrinsic atopic dermatitis

AD can be categorized into extrinsic and intrinsic AD, which account for approximately 80% and 20% of disease carriers, respectively. Extrinsic AD is characterized by elevated levels of total IgE and allergen-specific IgE, a high percentage of eosinophils, and a family history of atopic disease. It is the primary subtype associated with skin barrier dysfunction and frequently with a high rate of *FLG* (encoding filaggrin) gene mutation. Patients with extrinsic AD often exhibit allergies to proteins or food [38, 39]. In contrast, patients with intrinsic AD typically have normal IgE levels and lack personal or family histories of atopic conditions [38, 39]. It tends to present later in life, is generally less severe, and often has normal skin barrier function. This subtype is frequently associated with metal allergies, possibly linked to suprabasin deficiency [38, 39]. While both intrinsic and extrinsic subtypes exhibit strong Th2 activation, intrinsic AD shows higher immune cell infiltration, possibly due to stronger activation of Th1 responses [29, 38–40]. This makes intrinsic AD more likely to develop into chronic AD.

#### Factors influencing manifestations of atopic dermatitis

The manifestations of AD vary with patient age. Infantile AD usually presents with a widespread distribution of acute lesions characterized by erythema, edema, epidermal detachment, and exudation. In childhood AD, eczema becomes progressively more chronic, with lighter colored erythema and dryness, as well as chronic mossy thickening of the skin [1]. It has also been shown that the peripheral blood phenotype of early childhood AD exhibits only Th2 cell expansion, with no other T cell subsets observed in the blood. In contrast, the blood of adult patients with AD shows increased Th22 and Th2 activation [40]. Therefore, we hypothesized that patients with AD might show symptoms of the acute phase in early childhood, such as erythema and edema, due to the mere presence of Th2 cell activation, whereas adults with AD might exhibit increased proliferation of the epidermis due to enhanced levels of Th22 cells that push the disease to the chronic phase. Thus, the manifestation of AD may be related to the infiltration of different T cell subtypes.

The prevalence and manifestations of AD differ across races. In the United States, the prevalence among Black children (19.3%) was slightly higher than that among White children (16.1%) [2]. In addition, the prevalence of AD is higher among Asians than among Caucasians, and Asian patients with AD primarily exhibit chronic manifestations such as epidermal thickness, elevated Ki67 levels, and widespread dyskeratosis [41]. Studies have shown that the Th17 and Th22 axes are significantly more activated in Asian patients than in those from Europe and America, and Asian patients with AD might have a mixture of AD and psoriasis according to the overall cytokine profile and atypical disease features (e.g., keratosis pilaris) [29, 34, 40]. Therefore, high IL-17 and IL-22 levels may be associated with chronic AD lesions in Asian patients.

## Molecular pathogenesis of atopic dermatitis

The pathogenesis of AD involves a complex interplay between inherent genetic susceptibility and diverse environmental triggers, where intrinsic predisposition increases the chance of skin barrier dysfunction, and external perturbation exploits this intrinsic defect and causes further damage. These intrinsic and extrinsic factors converge to facilitate allergen penetration and microbial invasion, leading to dysregulation of immune reactions. Emerging evidence has underscored the significant contribution of redox imbalances in driving the molecular pathogenesis for perpetuating severity.

### Genetic background of atopic dermatitis

Some patients with AD have a clear family history of the disease, as significantly higher concordance has been demonstrated in identical twins than in fraternal twins [2]. Among the various genetic factors associated with AD, those associated with FLG are considered to have a leading genetic role, affecting 30%–50% of white patients with AD [2]. Specifically, *FLG* encodes FLG that plays a vital role in producing lipids and NMF, which act as a ‘mortar’ to anchor keratinocytes in the stratum corneum; degradation of FLG produces several key metabolites, including free amino acids, deiminated polycarboxylic acids, and urocanic acid, which collectively constitute the NMF [11]; loss of *FLG* initially causes keratinocyte cytoskeleton contraction, impairing the delivery of lamellar body contents and thereby disrupting the formation of the lipid barrier; additionally, reduction of urocanic acid, a FLG-derived metabolite, leads to increased stratum corneum pH that activates serine proteases, further exacerbating epidermal peeling and scaling [11]. Mutations in *FLG* disrupt this process, leading to TEWL and skin dryness [42]. A weakened skin barrier in patients with AD further promotes pathogen colonization, further impairing the skin barrier [6]. A pioneering study enrolling 16 pediatric patients meeting the Hanifin-Rajka diagnostic criteria in India identified multiple loss-of-function mutations in *FLG* (located on chromosome 1), such as 152276761T > C and 152276828G > A, which were present in 63% of the patients [43]. Other genetic variants involved in skin barrier formation also exist, e.g., *SPINK5* that encodes the serine peptidase inhibitor Kazal type 5, *LEKT1* encoding the lymphoepithelial Kazal-type-related inhibitor 1 [44–46], *FLG2* that encodes filaggrin 2, *HRNR* which encodes hornerin, and TCHH1 encodes trichohyalin [11]. In addition, genetic variants of *HLA* (encoding human leukocyte antigen) [8, 43] and *IL-13* (encoding interleukin 13) [8], which are associated with antigen presentation and inflammation, have been reported to play critical genetic predisposing roles. Although AD is often accompanied by asthma, children with asthma exhibit higher somatic mutation rates in their IgE sequences (7.2%), reflecting a more pronounced antigen-driven maturation process. In contrast, children with AD show a Somatic mutation rate of only 3.4%, which is less than half that observed in children with asthma [47].

**Fig. 1:**
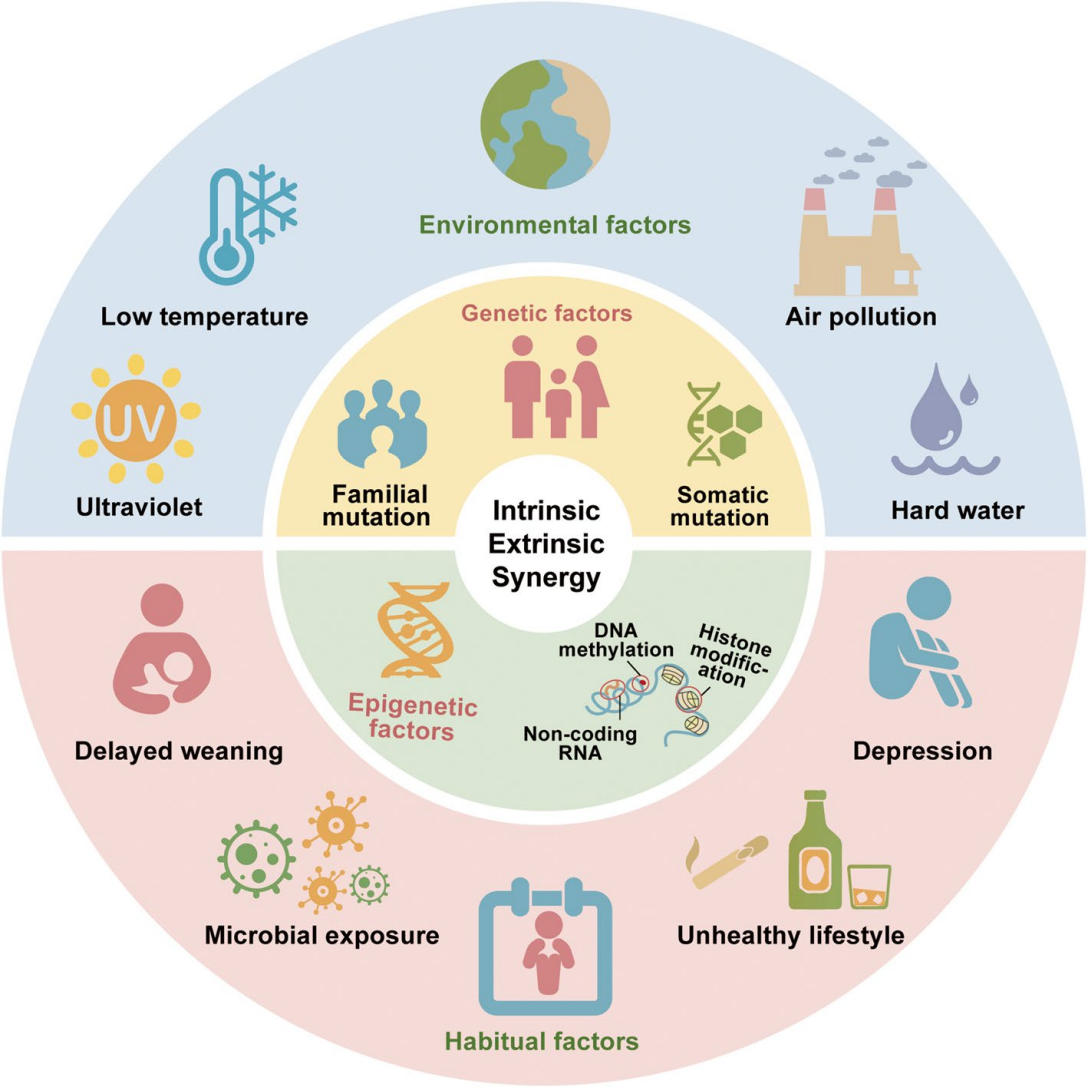
Intrinsic and extrinsic synergy in AD. Intrinsic factors comprise genetic and epigenetic components. Genetic factors include familial or somatic mutations, whereas epigenetic factors mainly involve DNA methylation, histone modifications, and non-coding RNAs. Extrinsic factors consist of environmental and lifestyle influences. Environmental factors include ultraviolet radiation, low temperatures, air pollution, and hard water, while lifestyle factors involve delayed weaning, microbial exposure, unhealthy behaviors, and psychological stress such as depression

Epigenetic modifications such as DNA methylation, histone modifications, and regulation of non-coding RNAs have been implicated in the onset of AD [48]. As examples of aberrant DNA methylation in patients with AD, increased methylation of S100A5 and decreased methylation of KRT6A and OAS2 were found in the epidermis of lesional skin [48]; 10 and 25 genes were identified to be hypo- and hypermethylated in CD4 + CLA + T cells of patients with AD, respectively [48, 49], and a differential methylation pattern was identified in *NLRP* between patients with AD and controls [48, 50, 51]. Several agents used to treat AD suppress the activity of histone deacetylase (HDAC). For example, HDAC inhibitors such as trichostatin A reduced the secretion of Th2 cytokines [52, 53]; butyric acid inhibited HDAC activity and thus reduced IL-6 levels [48, 54, 55]; and glucocorticoids inhibited the expression of TNFα and IL-8 by reducing HDAC activity [52, 56, 57]. Non-coding RNAs, especially microRNAs (miRNAs), play significant roles in the epigenetic regulation of AD. Differential expression of miRNAs, such as miR-21-3p, miR-130b-3p, miR-150-5p, and miR-1275, has been observed in AD, which function by regulating the methylation profiles of genes such as *ESR1, NDFIP2, ASB2,* and *TNRC6A* [48]. Other miRNAs such as let-7a-d, miR-375, miR-26a-5a, miR-21, miR-29b, miR-146a, and miR-155 are also differentially expressed in AD [52]. Fig. 1 shows the genetic and epigenetic factors that influence AD development.

### Environmental triggers of atopic dermatitis

In genetically or epigenetically predisposed individuals, the dysfunctional skin barrier interacts with environmental stressors to drive AD development [6]. Environmental factors play a vital role in the initial damages to the skin barrier, which once compromised activates the immune system, leading to further weakening of the barrier and a self-amplifying vicious cycle [11] (Fig. 1). External factors, such as low outdoor temperature, UV exposure, air pollution [2], and hard water, have been associated with the worsening of AD symptoms. It has been reported that AD is more common in regions with higher levels of industrialization, urbanization, and income [18], with the prevalence rate in industrialized nations having doubled over the past 30 years [18]. Exposure to high levels of PM2.5 during late pregnancy and early postnatal life has been closely associated with the subsequent development of AD in children [58]. In addition, delayed weaning in infants is associated with an increased risk of developing severe AD [2]. Microbial exposure is another key factor in AD pathogenesis. In recent years, decreased exposure to infections such as hepatitis and tuberculosis in early childhood has been linked to increased susceptibility to allergic diseases [6]. Certain lifestyle factors may protect against AD. For example, consuming unpasteurized farm milk in the first two years of life and direct contact with farm animals during pregnancy appear to lower the risk of developing AD [6]. Moreover, unhealthy lifestyles and psychological stress contribute to AD development [8, 59]. These findings suggest that early microbial exposure helps establish immune resilience and potentiates a reduced incidence of allergic diseases. Fig. 1 shows the environmental factors influencing AD.

### Immune dysregulation and cellular mechanisms in atopic dermatitis

**Fig. 2:**
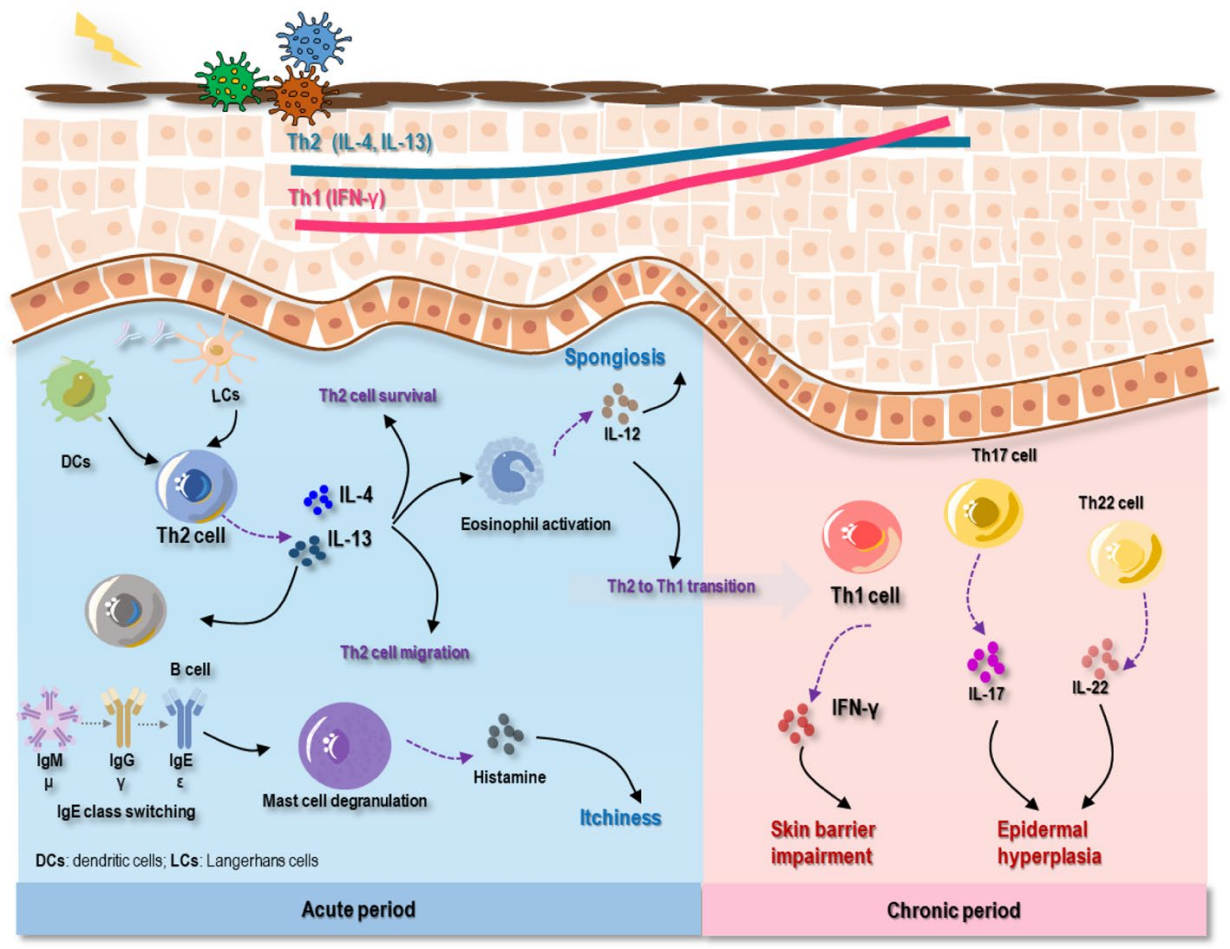
The pathogenesis of AD. In the acute phase of AD, external stimuli such as bacterial infection and allergens activate APCs, including DCs and LCs, which can further activate Th2 cells to secrete cytokines such as IL-4 and IL-13. These cytokines support Th2 cell survival and migration to lesion sites, promote eosinophil activation (which subsequently contributes to the Th2-to-Th1 transition), and induce B-cell IgE class switching, leading to mast cell degranulation. Activated eosinophils secrete IL-12, contributing to spongiosis, while degranulated mast cells produce histamine, causing itchiness in acute AD. In the chronic phase of AD, Th1 cells play the dominant role by secreting IFN-γ, which impairs the skin barrier. In addition, Th17 and Th22 cells are involved, producing IL-17 and IL-22 that enhance epidermal thickness. Abbreviations: APCs, antigen-presenting cells; DCs, dendritic cells; LCs, Langerhans cells; Th, T helper cell; IgE, immunoglobulin E

The pathogenesis of AD can be captured by acute and chronic phases and is orchestrated by a panel of immune-regulatory factors that involve multiple immune cells and cytokines. Among the various types of cells and molecules involved in the immune response, Th1, Th2, Th17, and Th22 cells play leading roles in dictating the AD phase and coexist in both the acute and chronic phases of AD. Specifically, Th2 cells play a dominant role in the acute stage, and Th1 cells play a role in the chronic phase, despite their concomitant incremental profiles (Fig. 2). Although the number of B cells is elevated in the blood of patients with AD, their precise mechanism of action remains unclear [8].

The main pathological causes priming the initiation of acute AD include skin barrier damage, immune abnormalities, and alterations in the skin microbiota. When the skin barrier is impaired, antigens pass through the epidermis and activate epidermal Langerhans cells (LCs) and dendritic cells (DCs) to activate Th2 cells, leading to the production of cytokines such as IL-4 and IL-13. These cytokines can further aid in the survival of Th2 cells by enhancing their migration to lesion sites. This results in the activation of eosinophils and contributes to class switching of the B cell immunoglobulin isotype, resulting in the production of IgE, which induces mast cell degranulation, histamine production, and consequently, pruritus [12, 60, 61]. Although IgE release is involved in AD pathogenesis, therapies targeting IgE have not shown significant clinical improvement, and the underlying mechanisms remain unclear [8]. Recent advances revealed an “itch-scratch” cycle during AD pathogenesis. The Th2-associated cytokines IL-4 and IL-13 not only mediate inflammation but also enhance neuronal sensitivity, and sensory neurons express receptors for type 2 cytokines [8]. Accordingly, blocking Th2-associated pruritogenic cytokines is effective in relieving itching [8].

The dominant role of Th2 cells in the immune response may be taken over by Th1 cells, leading to a transition from acute lesions to the chronic phase when cytokines secreted from Th2 cells stimulate the production of IL-12 by eosinophils [61–63]. During this phase transition, the number of Th1, Th17, and Th22 cells significantly increases, leading to the production of cytokines, such as IL-17 and IL-22, which collectively inhibit the terminal differentiation of keratinocytes while promoting their proliferation in the epidermis [12]. Moreover, as a negative immunoregulatory factor, the expression of IL-34 is reduced in AD [64]. Its deficiency can lead to the loss of LCs [64, 65]. This may impair epidermal antigen presentation, disrupt immune tolerance, and exacerbate the inflammatory response. This cytokine milieu establishes self-reinforcing circuits linking barrier disruption, immune activation, and neurocutaneous signaling, underpinning the multifaceted pathology of AD.

### Central cytokines and inflammatory pathways driving atopic dermatitis

Polarization of Th1/Th2/Th17/Th22 cells is controlled by the cytokines produced. While Th1 cells mainly generate IFN-γ, Th2 cells express IL-4/IL-13; while Th17 cells primarily secrete IL-17, Th22 cells produce IL-22. Dynamic regulation of these cytokines is critically involved in the transition from the acute to the chronic phase of AD [12, 29]. It has been shown that reduced production of Th2 cytokines IL-4 and IL-13 shifted the immune response toward Th1 dominance [35, 36]; and systemic administration of the Th1 cytokine IFN-γ during the acute phase suppressed Th2 development [66].

The Th1 cytokine IFN-γ exacerbates skin barrier dysfunction in AD by disrupting the fatty acid composition of ceramides, a critical component of the stratum corneum lipid matrix, thereby compromising epidermal integrity and increasing susceptibility to cutaneous infections commonly observed in patients with AD [67]. Furthermore, IFN-γ antagonizes IL-4-mediated signaling, leading to suppressed activation of Th2 lymphocytes and reduced IgE production, a hallmark of allergic inflammation in AD. This dual mechanism not only perpetuates barrier impairment but also induces keratinocyte apoptosis, resulting in weakened structural resilience of the skin and an amplified vicious cycle of inflammation [68]. The interplay between IFN-γ-driven Th1 responses and suppressed Th2 activity highlights complex immune dysregulation in AD, where shifts in cytokine dominance contribute to both pathological tissue remodeling and compromised antimicrobial defense mechanisms.

The Th2 cytokines IL-4 and IL-13 drive susceptibility to cutaneous infections in AD by suppressing the expression of AMPs such as β-defensins and cathelicidins, which are critical for defending against pathogens like Staphylococcus aureus commonly colonized in AD lesions [40]. This immunosuppressive effect on innate immunity is partially counterbalanced during chronic inflammation by elevated IL-17 and IL-22 levels, which restore AMP production and enhance epithelial defense mechanisms [40, 69]. Simultaneously, IL-4 and IL-13 exacerbate skin barrier dysfunction by down-regulating key structural proteins such as FLG and Lipids while aberrantly modulating sex steroid hormone synthesis through the induction of 3β-hydroxysteroid dehydrogenase 1, an enzyme that converts dehydroepiandrosterone to active androgens and estrogens [70, 71]. Hormonal dysregulation disrupts keratinocyte differentiation and lipid organization, leading to a permeable stratum corneum, increased TEWL, and enhanced allergen penetration. Collectively, these mechanisms highlight the dual role of Th2 cytokines in the perpetuation of immune suppression and structural fragility in AD.

IL-17 and IL-22 collectively disrupt epidermal homeostasis by inhibiting the terminal differentiation of keratinocytes, impairing the formation of a compact stratum corneum, and promoting aberrant epidermal hyperplasia, which manifests as thickened, hyperkeratotic skin lesions characteristic of chronic AD [12, 30, 60]. These cytokines, along with Th2-derived IL-4 and IL-13, act synergistically to suppress critical skin barrier proteins, including FLG, loricrin, and involucrin, while simultaneously downregulating lipid-metabolizing enzymes essential for maintaining the cornified envelope and lamellar lipid bilayers [12, 40]. This multi-cytokine crosstalk not only destabilizes the physical barrier by reducing keratinocyte cohesion and increasing TEWL but also amplifies immune dysregulation by facilitating allergen penetration and microbial colonization. Furthermore, IL-17 and IL-22 exacerbate inflammation-driven barrier defects by inducing pro-inflammatory mediators that perpetuate keratinocyte stress and apoptosis, whereas the IL-4/IL-13 pair reinforces this vicious cycle by suppressing antimicrobial peptide production and promoting Th2-polarized inflammation. Thus, the convergence of Th2, Th17, and Th22 pathways creates a self-sustaining inflammatory microenvironment in AD, where impaired barrier function and immune hyperactivation mutually reinforce disease chronicity, structural remodeling, and susceptibility to secondary infections.

Beyond the canonical cytokines, a network of immune mediators orchestrates the pathogenesis and progression of AD. For instance, thymic stromal lymphopoietin (TSLP), which is highly expressed by keratinocytes in skin lesions of patients with both acute and chronic AD, is recognized as a key molecule in the pathophysiology of AD. Specifically, TSLP induces a Th2 immune response by activating DCs and LCs, promoting the degranulation of mast cells, and supporting the growth of T cells. The serum level of TSLP is significantly higher in adult and pediatric patients with AD than in healthy individuals, and polymorphisms of the *TSLP* gene are strongly associated with an increased risk of AD progression [72]. In addition, non-lymphocytes secrete CCL17 and IL-25/IL-33 to activate intrinsic lymphocytes (ILCs) and Th2 cells. It has been reported that elevated CCL17 levels in peripheral blood were associated with the development of AD [70, 73]. IL-25, a member of the IL-17 family, has recently gained attention as an ‘alarmin indicating skin barrier damage [70]. IL-33 is highly expressed in the keratinocytes of patients with AD [74], which impairs the skin barrier by reducing FLG expression and contributes to itchiness by creating a mutually promoting vicious cycle with IL-31 [74]. In addition, Th2 cells secrete IL-5/IL-31 to promote disease progression [32], with IL-5 being related to the enhanced sensitivity of eosinophils to allergic inflammatory responses [75, 76]. IL-31 is a key pruritogen associated with itching [77, 78]. Moreover, as a negative immunoregulatory factor, the expression of IL-34 is reduced in AD [64]. Its deficiency can lead to the loss of LCs [64, 65]. This may impair epidermal antigen presentation, disrupt immune tolerance, and exacerbate the inflammatory response. This cytokine milieu establishes self-reinforcing circuits linking barrier disruption, immune activation, and neurocutaneous signaling, underpinning the multifaceted pathology of AD.

### Redox imbalance in AD: novel insights into pathogenic mechanisms

Cellular redox homeostasis dynamically balances oxidation and reduction, which is crucial for maintaining the physiological functionality of cells, and is implicated in the pathogenesis of various syndromes once disrupted. Reactive oxygen species (ROS), including short-lived radicals such as O_2_•^-^ and •OH and longer-lived molecules such as hydrogen peroxide (H_2_O_2_) and hypochlorous acid (HClO), play dual roles in a dose-dependent manner. Although excessive ROS levels cause DNA damage and induce cysteine oxidation and lipid peroxidation, physiological ROS levels are essential for signal relay. Antioxidant defense relies on enzymes such as superoxide dismutase (SOD) and catalase (CAT), alongside non-enzymatic thioredoxin (Trx) and glutathione (GSH) systems, to counteract overt oxidative stress [79–81].

Oxidative stress plays an important role in acute AD pathogenesis. Notably, children with acute AD exhibit significantly higher serum levels of lipid hydroperoxides and lower total antioxidant capacity [82]. Since lipid peroxidation is associated with the Fenton and Haber–Weiss reactions that are driven by OH•, O_2_^•−^ and H_2_O_2_ [83, 84], it is thus highly possible that acute AD is primarily caused by these reactive species potentiated by distorted electron transfer in the ETC and disrupted transition metal homeostasis. Of note, O_2_^•−^ and H_2_O_2_ are the primary reactive species generated during mitochondrial respiration when the leaky electrons are received by O_2_ [85–87]. O_2_^•−^ can be rapidly converted to H_2_O_2_ by SOD1 and SOD2 [84, 88, 89]. Through multiple mechanisms such as homolysis of H_2_O_2_ or an excited water molecule (H_2_O)*, H_2_O_2_ can be converted to OH· in vivo through reacting with certain transition metal ions such as Fe^2+^ [90]. OH· is the most reactive type of ROS that forms adducts with all four DNA bases to cause base loss and double/single strand breaks [91]. The damaging role of these reactive species in acute AD is evident as elevated levels of 4-hydroxy-2-nonenal (HNE), malondialdehyde (MDA), and 8-hydroxy-2’-deoxyguanosine (8-OHdG) have been observed in acute AD and are associated with disease severity [92], which are markers of DNA damage [93].

Interestingly, keratinocyte-derived ROS, whether originating from allergens or non-allergens, can induce Th2 inflammation in acute AD. It has been reported that molecules of the Th2 pathway such as kallikrein 5 (KLK5), proteinase-activated receptor 2 (PAR2), nuclear factor kappa-B (NF-κB), cytokines derived from epithelial cells such as TSLP, IL-25, IL-33, and neurogenic inflammatory molecules such as nerve growth factor (NGF) and calcitonin gene-related peptide (CGRP) were all up-regulated in normal human epidermal keratinocytes (NHEKs) treated with house dust mite allergen or non-allergen Like compound 48/80, the process of which was blocked by the anti-oxidant N-acetylcysteine [94]. Consistent with this, treating cells with pro-oxidants upregulates the expression levels of these biomarkers [94]. It is also worth noting that prenatal depression and anxiety increase the risk of developing neonatal AD by activating ROS signals [95].

Both genetic and environmental factors contribute to elevated ROS levels in acute AD. Clusterin, a sensitive sensor of oxidative stress, is elevated in the serum of children with acute AD, the levels of which are positively correlated with disease severity [96]. Genetic polymorphisms in anti-oxidant enzymes such as glutathione-S-transferase (GST) pose a high risk of developing AD in children [97]. Moreover, significantly reduced paraoxonase (PON) activity was observed in the serum of children with AD, the activity of which was negatively correlated with cellular ROS level [82]. In addition, mutation or loss of transmembrane protein 79 (Tmem79), an orphan transmembrane protein with sequence homology to microsomal GST, renders the body sensitive to ROS-induced damage and acute AD onset [98]. As for environmental factors, exposure to air pollutants such as ozone (O_3_) is associated with the development of several inflammatory skin diseases including acute AD [99]. O_3_ exposure increases transcript and protein levels of inflammasome complexes such as apoptosis-associated speck-like protein containing a CARD (ASC) and caspase-1, and promotes the secretion of IL-18 and IL-1β, where ROS plays a vital role in the activation of the complex [100]. Exposure to mold also increases the risk of acute AD by relaying ROS-activated signaling [101].

**Fig. 3:**
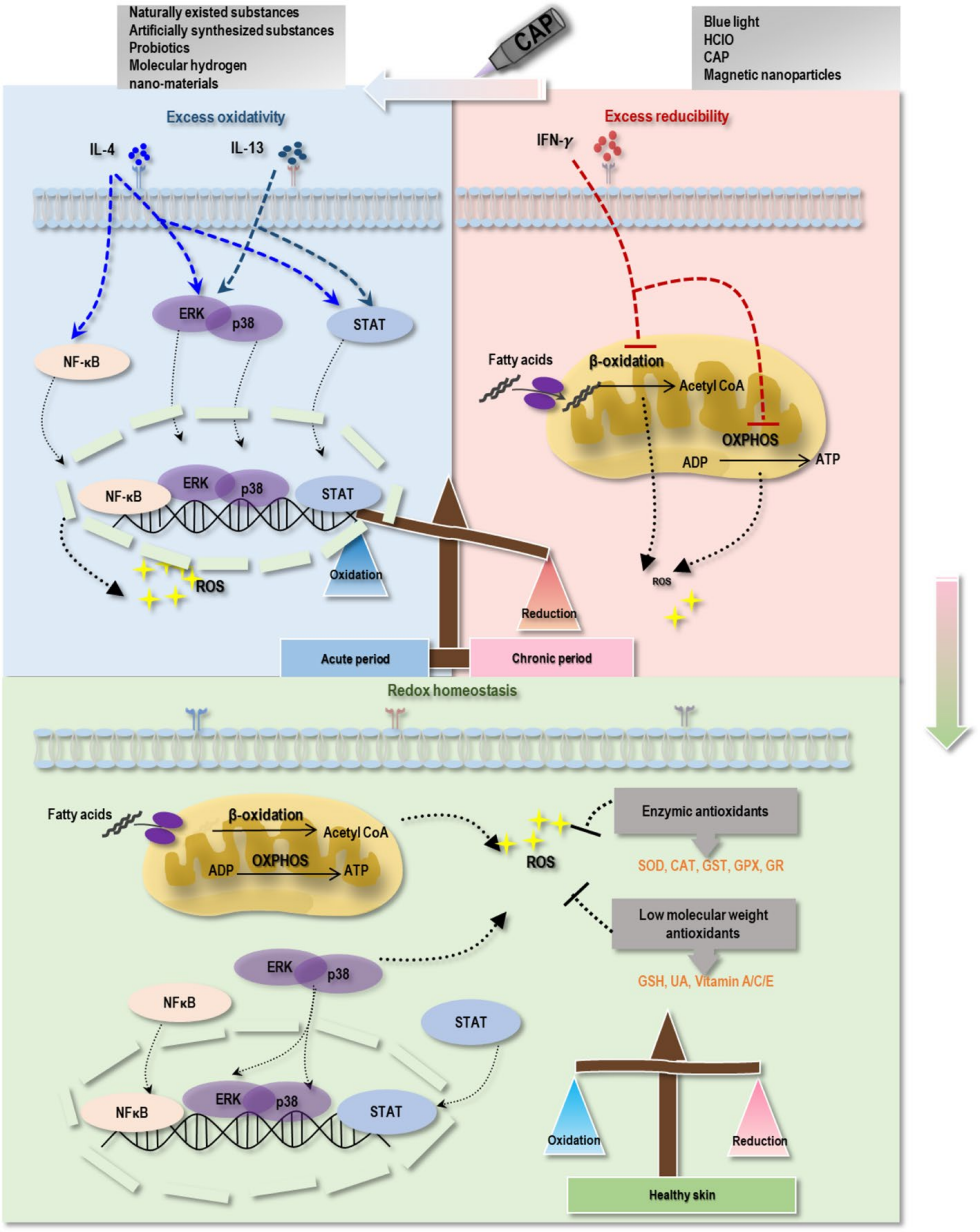
The wound healing model for AD and the pro-oxidative properties of CAP for treating chronic AD. Acute AD is characterized by oxidative stress, whereas chronic AD is characterized by reductive stress. In the acute phase, cytokines secreted by Th2 cells, such as IL-4 and IL-13, activate pathways including JAK/STAT, MAPK (p38, ERK), and NF-κB, which impose oxidative stress on cells (blue part). In the chronic phase of AD, IFN-γ secreted by Th1 cells predominates and suppresses fatty acid β-oxidation and OXPHOS in mitochondria, resulting in reduced ROS production (red part). In healthy skin, reductive substances—including enzymic antioxidants (SOD, CAT, GST, GPX, GR) and low-molecular-weight antioxidants (GSH, UA, vitamins A/C/E)—effectively neutralize ROS generated from signaling pathways such as MAPK (p38, ERK), JAK/STAT, NF-κB, as well as oxidative events occurring in mitochondria, including fatty acid β-oxidation and OXPHOS, thereby maintaining cellular redox homeostasis (green part). According to the proposed “wound healing model,” antioxidants can be used for treating acute AD, whereas pro-oxidative strategies, including CAP, can be applied to alleviate reductive stress in chronic AD, thereby restoring redox homeostasis or shifting chronic AD back to the acute stage, after which therapeutics effective for acute AD treatment can be applied. Abbreviations: ROS, reactive oxygen species; JAK, Janus kinase; STAT, signal transducer and activator of transcription; MAPK, mitogen-activated protein kinase; NF-κB, nuclear factor kappa-B; OXPHOS, oxidative phosphorylation; CAP, cold atmospheric plasma; SOD, superoxide dismutase; CAT, catalase; GST, glutathione-S-transferase; GPX, glutathione peroxidase; GR, glutathione reductase; GSH, glutathione; UA, uric acid

The molecular mechanisms linking ROS elevation and acute AD can be explained as follows (Fig. 3). ROS are generated within keratinocytes in response to the infiltration of inflammatory cells into the skin or in response to environmental stimuli. Excess ROS upregulates the expression of pro-inflammatory cytokines towards increased skin inflammation and impaired skin barrier function. In this process, excess ROS activates IκB kinase that helps translocate NF-κB to the nucleus to regulate the transcription of downstream genes, leading to increased TSLP and activated cutaneous LCs. LCs, once activated, migrate to lymph nodes where they present antigens to T cells, leading to the differentiation and proliferation of CD4^+^ T cells [102]. Consistent with this, treating NHEKs with IL-4 and IL-13 leads to high intracellular ROS levels, which exacerbate acute AD by activating the mitogen-activated protein kinase (MAPK) pathway and p38 and ERK signaling [103]. Some photochemicals with anti-oxidant effects or capable of inhibiting NF-κB and MaPK signaling can exert therapeutic effects in attenuating acute AD via inducing nuclear factor erythroid 2-related factor (Nrf2) expression [102]. In addition, supplementing keratinocytes with SOD reduces TNFα-induced ROS and inflammation through an NF-κB-dependent mechanism [104].

Although numerous studies have demonstrated the effectiveness of anti-ROS strategies in treating acute AD, several reports have proposed controlling chronic AD by elevating ROS levels. For instance, using novel in vivo dynamic nuclear polarization magnetic resonance imaging (DNP-MR), redox alterations in the skin of acute AD (lesions treated with Biostir AD ointment for 2 weeks) and chronic AD (lesions treated with Biostir AD ointment for 4 weeks) were monitored for the first time using a mouse model. The rate of free radical scavenging was lower in chronic AD mice than in acute AD mice, suggesting an increased reducing power from the acute to chronic AD phase. In addition, although more severe epidermal thickening existed in chronic mice, ROS were not enhanced [37]. These results collectively suggest that ROS levels are downregulated as AD progresses from the acute to the chronic phase.

Unlike most antioxidant treatment modalities, one study proposed that tissue hypoxia, namely hyperbaric oxygen therapy (HBOT) or the oxygen-carrying chemical perfluorodecalin (PFD), can also attenuate AD by inducing oxidative stress. Specifically, significantly attenuated disease symptoms were observed in a mouse model of chronic AD after HBOT or PFD treatment, with notable reduction in the levels of IL-17 and IFN-γ [105]. These findings suggest the possibility of treating chronic AD by increasing cellular ROS levels.

Given the important roles played by ROS during the acute and chronic phases of AD, we proposed a wound-healing model to capture its pathogenesis from the perspective of redox regulation (Fig. 3). In this model, patients with acute or chronic AD develop redox disequilibrium. While excess redox levels exist in the acute phase of AD, the reducing power is taken over when disease progression enters the chronic phase. Since the chronic phase of AD is associated with abnormal epidermal proliferation (e.g., epidermal thickening) and involves Th2/Th1/Th22/Th17 immune players, we considered this pathological condition to be a ‘psoriasis-like’ state. Therapeutically, it is possible to treat chronic AD by rewiring its reductive cellular environment back to redox homeostasis or elevating its redox level toward the acute state, followed by canonical therapeutics for acute AD, such as anti-oxidative agents.

A plethora of molecular evidence supports overt redox levels in acute AD (Fig. 3). Th2-type cytokines IL-4 and IL-13, which play an important role in ROS generation, significantly increased during the acute phase of AD. IL-4 treatment results in widespread proteomic thiol oxidation, among which protein tyrosine phosphatase (PTP) oxidation has been identified as the main redox-regulatory mechanism. Upon ligand activation, the IL-4 receptor generates ROS by activating NOX1 and NOX5L in a phosphoinositide 3-kinase (PI3K)-dependent fashion [106, 107]. Moreover, ROS produced by other cytokine receptors, such as erythropoietin, TNFα, or IL-3 receptor, also promoted IL-4 signaling [107]. IL-4 and IL-13 activated the downstream JAK/signal transducer and activator of transcription (STAT) signaling transduction to elicit oxidative stress and exacerbate inflammation [108, 109]. Thus, increased IL-4 levels during the acute phase of AD are associated with high ROS levels. In addition, activation of mast cells contributes to the generation of ROS such as superoxide and H_2_O_2_ [110]. Upon binding IL-4 to its receptor, the MAPK (p38, ERK) pathway is activated, which relays signals to PI3K for downstream activation of the STAT pathway [111, 112]. Besides, IL-4 enhanced the activation of the transcription factor NF-κB [113, 114]. These signalings, activated in response to cytokines secreted by Th2 cells, such as JAK/STAT, MAPK (p38, ERK), and NF-κB, collectively contribute to enhanced cellular ROS in the acute phase of AD.

This evidence also supports the existence of reductive stress in chronic AD (Fig. 3). Chronic AD is characterized by aberrant cell proliferation empowered by an overwhelming antioxidative response that counteracts increasing levels of ROS [115, 116]. It is well known that the mitochondrial electron transport chain utilizes an electron transfer reaction to generate ATP via OXPHOS, leading to the production of ROS [117]. Th1 cells take the dominant role in the chronic phase of AD that secrete IFN-γ to inhibit OXPHOS [118], possibly leading to increased reducibility. IFN-γ may also contribute to the cellular reductive stress by inhibiting fatty acid β-oxidation in the mitochondria [119, 120]. In addition, Th2 cells secreting IL-4 and IL-13 are reduced in chronic AD, leading to decreased ROS generation during this phase [30]. Though little has been reported on the regulation of IL-17 and IL-22 in the redox level of chronic AD, IL-17 inhibited fatty acid β-oxidation and exacerbated high-fat diet-induced hepatic steatosis [121]. Therefore, the dominant role of Th1 cells in the chronic phase may contribute to the reductive stress of chronic AD by suppressing mitochondrial OXPHOS and fatty acid β-oxidation. Other factors, such as decreased levels of cytokines secreted by Th2 cells, and the role of other cytokines such as IL-17 in suppressing fatty acid β-oxidation, may also be involved in the triggered reductive stress in chronic AD.

## Treatment strategy of atopic dermatitis

### Conventional treatment strategy for atopic dermatitis

**Fig. 4:**
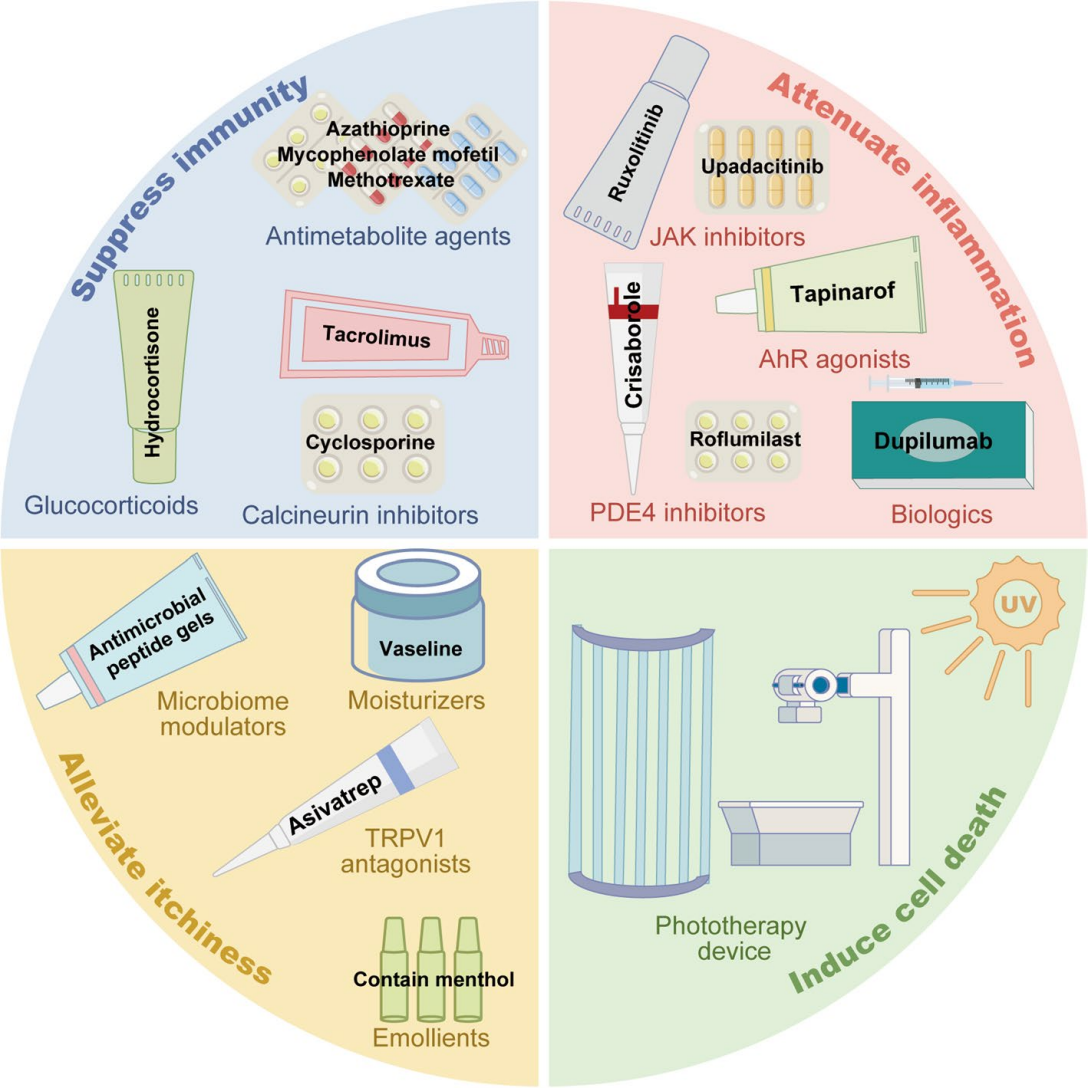
Categories of conventional AD therapies. Topical therapeutics for AD management can be roughly grouped into four categories based on their mechanisms of action: alleviating itchiness, suppressing immunity, attenuating inflammation, and inducing cell death. Agents in the first category include, for example, microbiome modulators, emollients, moisturizers, and TRPV1 antagonists. Therapeutics in the second category can be further divided into glucocorticoids, calcineurin inhibitors (e.g., tacrolimus, cyclosporine), and antimetabolite agents (e.g., azathioprine, mycophenolate mofetil, methotrexate). Therapeutics in the third category include JAK inhibitors, AhR agonists, PDE4 inhibitors, and biologics. Approaches that fall into the fourth category primarily involve UV phototherapy. Abbreviations: TRPV1, transient receptor potential vanilloid 1; JAK, Janus kinase; AhR, aryl hydrocarbon receptor; PDE4, phosphodiesterase 4; UV, ultraviolet

Before discussing AD therapeutics, it is important to emphasize the importance of educating patients about skin care and hygiene practices for AD management [122]. Patients should avoid reusing or sharing personal items that come in contact with the skin, such as towels, to minimize the risk of spreading bacteria, fungi, or other microbes. The skin should be kept clean without excessive washing or harsh cleansers, as they May damage the skin barrier. To prevent contamination, clean containers and tools should be used when applying treatments. French guidelines for AD recommend the adoption of short, lukewarm baths or showers using cleansing products free from allergens and irritants, with a pH between 5 and 6 [123]. Additionally, patients should maintain a clean environment to reduce allergens such as dust and pollen. If skin infections occur, appropriate antimicrobial treatment should be promptly administered. Reducing exposure to allergens is vital for preventing AD flares and worsening of symptoms. Patients should avoid known allergens, such as dust mites, pollen, and pet dander. Wearing masks and hats outdoors can help reduce exposure. Dietary adjustments are also advised, and patients should be cautious about food choices and avoid known or potentially allergenic foods [122]. Therapeutics for AD can be categorized into four main groups (Fig. 4): repair of the damaged skin barrier, suppression of immunity, inhibition of inflammation, and induction of cell death.

#### Drugs for restoring the damaged skin barrier

The treatment options fall into the first category and include emollients, moisturizers, microbiome modulators, and TRPV1 antagonists. As a primary preventive approach, emollients help restore the skin barrier before the emergence of AD symptoms [3]. Anti-itching emollients, including menthol, polidocanol, and lidocaine, provide local anesthetic effects that ease itching by reducing nerve sensitivity [2]. Moisturizers contain urea, bath oils, and gentle cleansers, which can thus help maintain skin hydration and alleviate dryness and itching [2, 124]. Furthermore, compared to a commonly used oil-in-water emulsion containing glycerin but without physiological lipids, a topical multivesicular emulsion containing physiological lipids and glycerin not only alleviates itching but also improves skin barrier function and reduces skin sensitivity [125]. Microbiome modulators such as antimicrobial peptide gels can inhibit harmful microbes and support a balanced skin microbiome [13, 126]. TRPV1 antagonists, such as asivatrep, can reduce itch sensitivity by inhibiting TRPV1 receptor activity in nerve endings [127].

#### Drugs for suppressing immunity

The second category of therapeutics for AD includes immunosuppressants such as glucocorticoids (e.g., hydrocortisone and clobetasol propionate), calcineurin inhibitors (e.g., tacrolimus and cyclosporine), and antimetabolite agents (e.g., azathioprine, mycophenolate mofetil, and methotrexate). Immunosuppressants reduce inflammation by decreasing pro-inflammatory cytokines [2, 128]. Topical glucocorticoids are the first line of treatment for relieving flare-ups in patients with AD [129–131]. Tacrolimus has been used topically for more than 20 years to treat AD, and its efficacy and safety have been supported by both short- and long-term clinical trials [132]. Cyclosporine is widely employed as both a short-term treatment and maintenance therapy for AD [2, 7]. Current guidelines generally recommend cyclosporine as the first-line systemic treatment for AD during pregnancy [133]. Azathioprine, a purine analog, inhibits DNA synthesis and suppresses the growth of rapidly dividing cells, such as T and B lymphocytes [134], by targeting glutamine phosphoribosyl amidotransferase (GPAT) and inosine monophosphate dehydrogenase (IMPDH) or by being incorporated into DNA/RNA [134]. Studies have shown that azathioprine can significantly reduce the disease severity of patients with AD, and thus remains a widely used treatment option for moderate-to-severe AD cases despite its undesirable tolerance [135, 136]. Some guidelines suggest azathioprine as a potential treatment option for managing AD in pregnant women [133]. Mycophenolate mofetil, a prodrug of mycophenolic acid (MPA), functions by inhibiting the rate-limiting enzyme IMPDH during purine synthesis to retard the proliferation of T and B cells that largely rely on this pathway for growth [137]. Although mycophenolate mofetil has been demonstrated to be effective in treating refractory AD cases [138], monitoring for potential infections is required for its long-term use [138]. Methotrexate, a folate antagonist, acts by inhibiting dihydrofolate reductase (DHFR, an enzyme required for converting dihydrofolate to tetrahydrofolate), thereby blocking DNA and RNA synthesis [139]. Methotrexate has demonstrated effectiveness in treating AD, with sustained benefits observed even after discontinuation of therapy [140]. Methotrexate is typically administered orally, and a Phase III clinical trial is underway in Europe to evaluate its clinical efficacy and safety through subcutaneous injection (starting at 20 mg/week) for the treatment of adult AD [141]. Novel immunosuppressive therapies are currently under development. The OX40-OX40L co-stimulatory pathway, which is critical for pathogenic T-cell activation, differentiation, and memory formation in AD, has emerged as a novel signaling axis. Therapies including monoclonal antibodies such as telazorlimab and rocatinlimab, and oral agents such as the S1P receptor modulator etrasimod, are currently undergoing clinical trials, and complementary strategies under investigation include enhancing the functions of regulatory T cells using IL-2 receptor agonists, developing bispecific antibodies, or utilizing multispecific nanobodies [8]. In addition, a phase II clinical trial of rilzabrutinib, an oral small-molecule Bruton's tyrosine kinase inhibitor, found that although there was no significant difference in the overall efficacy between the treatment and placebo groups in patients with AD, rilzabrutinib demonstrated rapid and consistent improvement in skin itch symptoms [142].

#### Drugs for inhibiting inflammation

Treatment modalities that fall into the third category include JAK inhibitors, AhR agonists, PDE4 inhibitors, and biologics. Cytokines, such as IL-4, IL-13, IL-31, and TSLP, rely on the JAK/STAT pathway for activation [143]. JAK inhibitors, including delgocitinib and gusacitinib that target pan-JAK, baricitinib and ruxolitinib target JAK1 and JAK2, tofacitinib targets JAK1 and JAK3, abrocitinib and upadacitinib target JAK1, block JAK signaling to help control AD-related inflammation [2, 8, 144, 145]. Approved JAK inhibitors include topical ruxolitinib, as well as oral baricitinib, abrocitinib, and upadacitinib [2, 8, 13, 145]. Two phase III clinical trials (TRuE-AD1 and TRuE-AD2) evaluating ruxolitinib cream monotherapy in adults and adolescents with mild to moderate AD demonstrated that ruxolitinib cream relieved skin pain within 12 h, significantly improved AD-related symptoms and quality of Life within 2weeks, and Maintained its efficacy for up to 52weeks [146]. Upadacitinib is superior to dupilumab in restoring the fungal microbiome in patients [147]. A multicenter retrospective study conducted in Italy confirmed that upadacitinib exhibits favorable short- and long-term efficacy across a broad range of AD phenotypes, including both typical (flexural) and atypical forms such as nummular eczema, erythrodermic AD, and prurigo nodularis-like presentations [148]. Upadacitinib is both safe and effective in smokers, with no significant difference compared with non-smokers [149]. AhR agonists such as Tapinarof (at concentrations of 0.5% or 1%) can support skin barrier gene expression and regulate Th2 cytokines by activating the AhR pathway, leading to reduced inflammatory oxidative damage and improved AD symptoms [2, 13, 143, 150]. A Maximal usage trial evaluated the safety and pharmacokinetic profile of 1% tapinarof cream applied once Daily in children and adolescents with extensive AD. The patients had a mean body surface area of 42.8%. The results demonstrated that tapinarof exhibited a favorable safety profile and pharmacokinetic characteristics in children and adolescents aged 2 years and older with extensive AD [151]. In the 2023 and 2024 guidelines for the management of adult AD published by the American Academy of Dermatology, Tapinarof cream is strongly recommended for adults with moderate to severe AD, with a high level of evidence [152]. PDE inhibitors have emerged as important therapeutic agents for the treatment of AD. Topical PDE4 inhibitors such as 2% crisaborole can reduce pro-inflammatory cytokines in AD by increasing cyclic adenosine monophosphate (cAMP) levels [2, 13, 153]. Systemic PDE4 inhibitors, such as the oral intake of roflumilast, are considered effective for AD treatment [154]. Roflumilast is also a topical formulation, and a 0.15% roflumilast cream is strongly recommended for adults with mild to moderate AD, with a high level of evidence [152]. Additionally, the selective PDE4B/D inhibitor orismilast has been clinically evaluated [155]. Results from the phase IIb ADESOS trial showed that a significantly higher proportion of patients receiving oral orismilast achieved IGA 0/1 with ≥ 2-point improvement as compared with placebo, suggesting its potential as a convenient and effective oral treatment for moderate-to-severe AD [155]. Biologics act by targeting key inflammatory mediators such as monoclonal antibodies against IL-4, IL-13, IL-22, IL-31, IL-33, and IL-18 [2, 8, 13, 144]. Among these, dupilumab is the only biologic agent specifically targeting the IL-4Rα and has been approved by the FDA for treating patients with moderate-to-severe AD aged 6 months and older; it was also approved in China in June 2020 [8, 156]. Dupilumab acts by inhibiting IL-4 and IL-13 signaling pathways, thereby reducing inflammation [157]. Clinical evidence supports the efficacy and safety of dupilumab in various age groups. A phase III placebo-controlled trial demonstrated significant improvements in caregiver-reported symptoms and quality of Life in children aged 6months to 5 years with moderate-to-severe AD [158]. Furthermore, a phase IV open-label study in adolescents and adults (≥ 12 years) showed that 16 weeks of dupilumab treatment restored both lesional and non-lesional skin barrier functions independent of the FLG genotype [159]. In addition, a multicenter retrospective study spanning 35 Italian dermatology centers reported a five-year drug survival rate of 81.47% for dupilumab with low discontinuation rates due to ineffectiveness or adverse events, indicating favorable long-term efficacy and tolerability [160]. Moreover, a systematic review suggested that dupilumab may be a suitable treatment option for women during pregnancy and breastfeeding [133]. Monoclonal antibodies that target IL-13 include tralokinumab and lebrikizumab. Tralokinumab has been approved by the FDA for the treatment of moderate-to-severe AD in adults [8, 161–166]. A systematic review and meta-analysis evaluating its real-world effectiveness and safety confirmed that tralokinumab demonstrated strong efficacy and favorable tolerability, with most patients experiencing significant clinical improvement and a low incidence of adverse events [164]. Furthermore, a clinical study has shown that tralokinumab significantly improves head and neck AD, particularly by alleviating skin discomfort and reducing the emotional burden caused by visible skin lesions [165]. Other biological agents include fezakinumab, which targets IL-22; nemolizumab, which targets IL-31; and etokimab, which targets IL-33. Fezakinumab exerts its effects by inhibiting IL-22 activity, and its efficacy has been demonstrated in a randomized double-blind placebo-controlled trial [167]. Lebrikizumab, approved by the FDA and recommended by the American Academy of Dermatology, can be used for treating moderate-to-severe AD in adults and adolescents aged 12 years or older [152, 168, 169]. An open-label, Phase IIIb clinical trial of lebrikizumab conducted in patients with moderate-to-severe AD and skin of color (Fitzpatrick skin types IV to VI) demonstrated that the drug effectively improved the signs and symptoms of AD, while maintaining a favorable safety profile [169]. Lebrikizumab has emerged as an effective and well-tolerated short-term alternative treatment option for patients with moderate-to-severe disease who respond inadequately to tralokinumab [163]. Additionally, in patients refractory to prior upadacitinib therapy, lebrikizumab demonstrated significant mid-term efficacy and favorable tolerability [168]. Nemolizumab, a humanized monoclonal antibody targeting the IL-31Rα, has shown significant efficacy in relieving pruritus and has been approved by both the FDA and the Japanese regulatory authorities [8, 170]. A Phase III clinical trial evaluated the efficacy and safety of nemolizumab in children aged 6–12 years with moderate-to-severe AD accompanied by intense pruritus over 68 weeks, and demonstrated substantial improvements in itch severity, AD symptoms, and quality of life [171]. Furthermore, the American Academy of Dermatology strongly recommends the use of nemolizumab in combination with topical therapy for adults with moderate-to-severe AD based on moderate-to-high-quality evidence [152]. Etokimab blocks IL-33 and its efficacy has been confirmed in a proof-of-concept clinical trial [172]. While these biologics have demonstrated promising efficacy by targeting well-characterized interleukins, emerging cytokines, such as IL-18, are also gaining attention as potential therapeutic targets, although current data remain limited [8, 173].

#### Drugs for inducing cell death

The last category refers to UV phototherapy, which induces DNA damage and triggers apoptosis in keratinocytes, T cells, and LCs [174, 175]. UV phototherapy is considered a suitable last resort for patients with steroid-resistant AD or those with systemic adverse outcomes, with narrow-band UVB (311–313 nm) and medium-dose UVA1 (340–400 nm) being effective options [2, 7].

Among these, emollients and moisturizers can only be used to alleviate symptoms such as itchiness. Omiganan, a microbiome modulator, corrects dysbiosis without any other noticeable clinical benefits [176]. The remaining treatments, although helpful for disease control, are typically accompanied by unavoidable side effects. For example, long-term use of immunosuppressants such as corticosteroid may damage the skin barrier or cause resistance [7, 16]; JAK inhibitors may cause side effects such as acne, nausea and headaches, gastrointestinal disturbances and thromboembolic events [2, 13, 145, 177], as well as an increased risk of infections, particularly viral infections, which are higher than those associated with dupilumab [178]; AhR agonists may cause side effects such as folliculitis, nasopharyngitis, contact dermatitis, headache, pruritus, and influenza-like symptoms [176]; PDE4 inhibitors may lead to headache, abdominal pain, depression, weight loss, nausea, diarrhea, vomiting, nasopharyngitis, and upper respiratory tract infections [179]; TRPV1 antagonists have been linked to side effects such as nasopharyngitis, hives, burning sensations, and runny nose [180]; biologics may cause dupilumab-associated ocular surface disease (DAOSD) and pityriasis versicolor, psoriasiform lesions [14, 15, 177, 181]; UV phototherapy imposes a risk of developing skin cancers [182]. Canonical therapeutics for AD treatment are listed in Table 3.

**Table 3:** Conventional therapeutics for AD

Drugs	Category	Mechanism type	Detailed mechanism	Development stage	Reference
Asivatrep	TRPV1 antagonist (Synthetic small molecule)	Repairing damaged skin barrier	•Block TRPV1-mediated Ca^2+^ influx to reduce neuropeptide release •Relieve pruritus and disrupt the neurogenic itch–scratch–skin damage cycle •Promote skin barrier protein expression	Clinical trial (NCT02757729: Phase II)	[127]
Hydrocortisone	Glucocorticoid (Synthetic small molecule)	Suppressing immunity	•Bind cytoplasmic GR •Translocate to the nucleus •Upregulate anti-inflammatory cytokines and downregulate pro-inflammatory cytokines •Suppress immune cell proliferation	Market approval	[2]
Clobetasol propionate	Glucocorticoid (Synthetic small molecule)	Suppressing immunity		Market approval	[2]
Tacrolimus	Calcineurin inhibitor (Synthetic small molecule)	Suppressing immunity	•Bind FKBP12 and form a complex that inhibits calcineurin •Prevent NFAT dephosphorylation and nuclear translocation •Downregulate pro-inflammatory cytokines •Suppress T cell activation and proliferation	Market approval	[132]
Cyclosporine	Calcineurin inhibitor (Synthetic small molecule)	Suppressing immunity	•Bind cyclophilin and form a complex that inhibits calcineurin •Prevent NFAT dephosphorylation and nuclear translocation •Downregulate pro-inflammatory cytokines •Suppress T cell activation and proliferation	Market approval	[2]
Azathioprine	Purine synthesis inhibitor (Synthetic small molecule)	Suppressing immunity	•Metabolize to 6-MP •Inhibit IMPDH and GPAT •Block purine nucleotide synthesis •Incorporate into DNA/RNA to disrupt nucleic acid synthesis •Suppress immune cell proliferation	Off-label use	[134]
Mycophenolate mofetil	Purine synthesis inhibitor (Synthetic small molecule)	Suppressing immunity	•Metabolize to MPA •Inhibit IMPDH •Reduce purine synthesis •Suppress immune cell proliferation	Off-label use	[138]
Methotrexate	Folate antagonist (Synthetic small molecule)	Suppressing immunity	•Inhibit DHFR •Block the reduction of DHFR •Impaired DNA/RNA •Suppress immune cell proliferation and activation	Off-label use	[140]
Delgocitinib	Pan-JAK inhibitor (Synthetic small molecule)	Inhibiting inflammation	•Inhibit JAK activation triggered by cytokines such as IL-4 and IL-13 binding to their receptors •Prevent downstream STAT protein phosphorylation and nuclear translocation •Downregulate pro-inflammatory cytokines	Market approval	[145]
Gusacitinib	Pan-JAK inhibitor (Synthetic small molecule)	Inhibiting inflammation		Clinical trial (NCT03654755: Phase 2; NCT03531957: Phase 2)	[145]
Baricitinib	JAK1 & JAK2 inhibitor (Synthetic small molecule)	Inhibiting inflammation		Market approval	[145]
Ruxolitinib	JAK1 & JAK2 inhibitor (Synthetic small molecule)	Inhibiting inflammation		Market approval	[145]
Tofacitinib	JAK1 & JAK3 inhibitor (Synthetic small molecule)	Inhibiting inflammation		Market approval; Off-label use	[145]
Abrocitinib	JAK1 inhibitor (Synthetic small molecule)	Inhibiting inflammation		Market approval	[145]
Upadacitinib	JAK1 inhibitor (Synthetic small molecule)	Inhibiting inflammation		Market approval	[145]
Tapinarof	AhR agonist (Synthetic small molecule)	Inhibiting inflammation	•Bind to AhR •Translocate to nucleus and complex with ARNT •Downregulate pro-inflammatory cytokines	Market approval	[150]
Crisaborole	PDE4 inhibitor (Synthetic small molecule)	Inhibiting inflammation	•Inhibit PDE4 activity •Increase intracellular cAMP levels •Activate PKA •Modulate the activity of transcription factors like NF-κB •Suppress the production of pro-inflammatory cytokines	Market approval	[153]
Roflumilast	PDE4 inhibitor (Synthetic small molecule)	Inhibiting inflammation		Market approval	[154]
Dupilumab	IL-4Rα monoclonal antibody (Biologic)	Inhibiting inflammation	•Block IL-4Rα •Inhibit IL-4 and IL-13 signaling •Suppress Th2-mediated immune inflammatory responses	Market approval	[156]
Tralokinumab	IL-13 monoclonal antibody (Biologic)	Inhibiting inflammation	•Block IL-13 signaling •Suppress Th2-mediated immune inflammatory responses	Market approval	[23]
Lebrikizumab	IL-13 monoclonal antibody (Biologic)	Inhibiting inflammation		Market approval	[161]
Fezakinumab	IL-22 monoclonal antibody (Biologic)	Inhibiting inflammation	•Block IL-22 signaling •Suppress Th22-mediated immune inflammatory responses	Clinical trial (NCT01941537: Phase II)	[167]
Nemolizumab	IL-31 monoclonal antibody (Biologic)	Inhibiting inflammation	•Inhibit IL-31 signaling •Suppress Th2-mediated immune inflammatory responses •Reduce pruritus and break the itch-scratch vicious cycle	Market approval	[170]
Etokimab	IL-33 monoclonal antibody (Biologic)	Inhibiting inflammation	•Inhibit IL-33 (an alarmin) •Block its binding to the ST2 •Downregulate pro-inflammatory cytokines such as IL-4 and IL-13	Clinical trial (NCT03533751: Phase II)	[172]
UVA1 (340–400 nm)	Phototherapy (Light)	Inducing cell death	•Induce DNA/RNA damage to trigger apoptosis	Off-label use	[7]
UVB (311–313 nm)	Phototherapy (Light)	Inducing cell death		Off-label use	[7]

*Abbreviations*: *TRPV1* transient receptor potential vanilloid 1, *CGRP* calcitonin gene-related peptide, *GR* glucocorticoid receptor, *FKBP12* FK506-binding protein 12, *NFAT* nuclear factor of activated T cells, *6-MP* 6-mercaptopurine, *IMPDH* inosine monophosphate dehydrogenase, *GPAT* glutamine phosphoribosyl amidotransferase, *MPA* mycophenolic acid, *DHFR* dihydrofolate reductase, *JAK* Janus kinase, *AhR* aryl hydrocarbon receptor, *ARNT* aryl hydrocarbon receptor nuclear translocator, *PDE4* phosphodiesterase 4, *PKA* protein kinase A, *cAMP* cyclic adenosine monophosphate, *IL-4Rα* IL-4 receptor alpha, *Th* T helper cell, *ST2* suppression of tumorigenicity 2

Summarily, the current obstacles to AD control largely rely on the management of the chronic phase because of the complex immune responses involved in this phase. One possible solution is to identify a set of key biomarkers for a patient to determine their response profile to existing therapeutics [3]. More importantly, proposing a pathological development model based on molecular profiles that characterize the acute and chronic phases of AD may help identify possible solutions for the treatment of chronic AD.

### Current redox modulating strategies for treating atopic dermatitis

#### Anti-oxidant strategies for treating acute atopic dermatitis

Various agents that demonstrate therapeutic efficacy against acute AD, including natural phytochemicals, nucleotides, polyamines, minerals, organic salts, and polyunsaturated fatty acids, are considered to have antioxidant effects. Natural phytochemicals such as alkaloids, flavonoids, and terpenoids can treat acute AD by exerting antioxidant effects [102]. These include alkaloids such as piperine, pseudoephedrine, magnoflorine (MAG), indirubin, esculetin, peiminine (PMN), tryptanthrin (TR) [183], flavonoids such as liquiritigenin, naringenin, diosmetin, baicalein, quercetin, puerarin, formononetin, chrysin, chamaejasmine, sulfuretin [183, 184], and triterpenoids such as ursolic acid (UA) [185]. Nicotinamide mononucleotide (NMN) is a naturally occurring nucleotide with antioxidant ability that has been proposed to alleviate AD-like symptoms by blocking ROS-mediated activation of the JAK2/STAT5 pathway [186]. Natural polyamines spermidine and spermine ameliorate swelling, edema, hemorrhage, and hyperkeratosis in AD-like skin lesions by inhibiting ROS production [187]. Furthermore, fish oil enriched with omega-3 PUFAs such as eicosapentaenoic acid (EPA) and docosahexaenoic acid (DHA) has demonstrated antioxidant and anti-inflammatory properties. Prenatal supplementation with omega-3 long-chain polyunsaturated fatty acids (n-3 LCPUFA), particularly in pregnant women with the TT genotype of the cyclooxygenase 1 (COX1) pathway genetic variant, is associated with a reduced risk of AD in offspring [188]. Trilinolein, an ester derived from glycerol and linoleic acid (an ω−6 PUFA), has shown promising anti-oxidant and anti-inflammatory potential [189]; and accordingly, a clinical trial (NCT06463353) aiming at exploring the possible benefits of Trilinolein cream in treating AD has been planned (Table 4). Tourmaline, which contains trace elements such as V, Cr, Zr, Mn, Ti, Sr, and Ga, is effective in alleviating a wide range of inflammatory and skin diseases, including AD, by emitting infrared rays and negative ions that lower the redox level in AD-carrying mice [190]. In addition, oxidase inhibitors such as diphenyleneiodonium (DPI), which suppress NOX, may exert a therapeutic effect against acute AD by reducing cellular ROS levels and the corresponding DNA damage [191]. Artificially synthesized substances, such as sodium thiosulfate (STS), known for their anti-oxidant and anti-inflammatory properties, were found to improve skin lesions in acute AD in vivo by reducing epidermal thickness, scratching time, inflammatory cytokine expression, dermal inflammatory cell infiltration, and ROS production [192].

**Table 4:** Ongoing clinical trials for anti-oxidative AD therapeutics

Drugs	NCT number	Phase	Mechanism	Status	Interventions	Patient	Sponsor
Probiotics	NCT05286047	NA	Repairing damaged skin barrier	Recruiting	Dietary supplement of bifidobacterium longum CCFM1029	Child	Glac Biotech Co., Ltd; China
Probiotics	NCT06230991	NA	Repairing damaged skin barrier	Not recruiting	Dietary supplement of SIM05 (a blend of 3 probiotics belonging to Bifidobacterium and Lactobacillus strains and 3 prebiotics)	Adult	Chinese University of Hong Kong; China
Probiotics	NCT06584552	II	Repairing damaged skin barrier	Not recruiting	Dietary supplement of lactobacillus paracasei LPB27	Child	The University of New South Wales; Australia
Probiotics	NCT06474728	NA	Repairing damaged skin barrier	Recruiting	Emollient containing postbiotic saccharomycess and lactobacillus	Child	Children's Hospital of Fudan University; China
Polyunsaturated fatty acids	NCT06463353	NA	Repairing damaged skin barrier	Not recruiting	Trilinolein cream	Child, Adult	Shanghai Yueyang Integrated Medicine Hospital; China

*Abbreviation*: *AD* atopic dermatitis

Probiotic strains, including lactic acid bacteria, possess anti-oxidant properties [193, 194] and their efficacy in treating AD has been explored both preclinically and clinically [195, 196]. Bifidobacterium longum is effective in increasing skin barrier function in patients with AD [197]. Topical application of the cell-free culture supernatant (CFS) of Bifidobacterium longum subspecies infantis (*B. infantis*) helped in maintaining the functionality of the skin barrier by increasing the activity of several anti-oxidant enzymes, including CAT and SOD, upregulating skin barrier-related genes such as *FLG*, *LOR*, *IVL*, *AQP3*, *TGM1,* and skin anti-microbial peptide genes such as *CAMP*, *hBD-2*, *hBD-3*, collectively leading to reduced ROS and MDA levels in H_2_O_2_-treated HaCaT cells [198]. A clinical trial (NCT05286047) was initiated to investigate the efficacy of Bifidobacterium longum CCFM1029 in treating children aged 1–3 years with AD. Another ongoing clinical trial (NCT06230991) aimed at evaluating the therapeutic potential of SIM05, a blend of three food-grade probiotics (belonging to Bifidobacterium and Lactobacillus strains) and three prebiotics, in managing adult patients with AD has also been planned and subjected to recruitment. In addition, Lactobacillus paracasei mixtures have demonstrated the potency of lowering cellular ROS levels [199], and oral administration of Lactobacillus paracasei KBL382 has been found to alleviate AD by modulating immune responses and gut microbiota [200]. A clinical phase II trial (NCT06584552) investigating the possible benefits of dietary supplementation with Lactobacillus paracasei in the treatment of early childhood eczema is ongoing. Additionally, Saccharomyces boulardii (CNCM I-745) can ameliorate ovalbumen-induced AD by modulating NF-κB signaling both in the skin and in the colon [201]. A clinical study (NCT06474728) evaluating the potential of an emollient containing Saccharomyces and Lactobacillus to reduce the risk of AD recurrence among children aged 0–6 years is currently underway. Table 4 summarizes the ongoing clinical trials investigating antioxidant-based therapies for AD.

Molecular hydrogen has been proposed as an effective approach for treating skin diseases [202], given its demonstrated efficacy in decreasing ROS generation in keratinocytes [203, 204]. For instance, mice fed hydrogen water (HW) showed improved acute AD symptoms, where the ROS level was significantly suppressed in the HW group compared to the purified water (PW) group, and the levels of TSLP and Th2 cytokines, as well as serum total IgE, were significantly lower in the HW group than in the control or PW groups [205].

Thus, ROS-scavenging hydrogels are promising for the treatment of acute AD. For instance, a hydrogel patch embedded with cerium dioxide nanoparticles (capable of scavenging ROS) reduced epidermal thickness and downregulated inflammatory cytokines in acute AD skin lesions, where cellular ROS levels were reduced [206]. In another study, a multifunctional hydrogel dressing with adhesion, self-repair, and anti-microbial properties was developed; by combining this hydrogel with the inhibitor of focal adhesion kinase (FAK), the compound effectively scavenged the intracellular ROS and reduced defective intercellular junctions and inflammatory responses induced by mechanical scratching [207]. By wrapping a hydrogel in a metal organic framework (MOF), that is, ZIF-8, Gel@ZIF-8 was fabricated, which substantially reduced epidermal thickness, decreased IgE levels, and mast cell infiltration, leading to decreased ROS and alleviated AD symptoms in mice with acute AD [208]. Fullerenes are nanomaterials that can act as radical scavengers. A fullerene derivative, fullerenol C60(OH)36, was synthesized and demonstrated to be a potential candidate compound for treating skin diseases, including acute AD, by reducing cellular ROS levels, decreasing the activity of MAPK signaling, dampening the expression of inflammation-related proteins, such as COX-2, heme oxygenase-1, and PGE2, and restoring skin barrier proteins, including FLG, involucrin, repetin, and loricrin, in keratinocytes [209]. CoO nanoenzymes with catalytic activities of SOD, CAT, and POD were synthesized and shown to protect HaCaT cells from inflammation and H_2_O_2_-induced ROS damage in MC903-induced AD mice [210].

#### Pro-oxidative strategies for treating chronic atopic dermatitis

It has been shown that the excitation of porphyrins (photoacceptors of blue light) by blue light (wavelength–400–500 nm) irradiation can lead to ROS formation in the skin and cultured human keratinocytes [211, 212]. A randomized clinical trial showed that 450 nm blue light improved itching and the patient-oriented scoring AD index (PO-SCORAD) in chronic AD [213]. However, the treatment outcome of blue laser was mild and could not improve other therapeutic indices such as the EASI, SCORAD, Investigator's Global Assessment (IGA), and Dermatology Life Quality Index (DLQI) [213]. The topical use of HClO (0.1%) or HClO hydrogel has been proposed as a therapeutic solution for treating chronic AD [214–216]. Also, 0.4%–0.5% sodium hypochlorite (NaClO) solution (also known as Dakin's solution) mitigated the inflammatory reaction and pruritus in chronic AD with minimized toxicity via inhibiting the NF-kB pathway, reducing T cell activation, attenuating mast cell activation and histamine release, reducing IgE production, inhibiting pro-inflammatory cytokines such as IL-1, IL-6, TNFα, IL-4, IL-13 and TSLP, and reducing intracellular calcium concentration in the dorsal root ganglion [217]. However, HClO and its derivatives have high cellular toxicity, and adverse effects such as temporary burning or stinging at the application site are unavoidable.

### Emerging redox modulating strategies for treating atopic dermatitis

CAP, which belongs to the fourth state of matter, exhibits several unique properties that make it feasible for biomedical applications, including its multimodal nature and selectivity against transformed cells. It is produced when air or an inert gas, single or mixed, is partially ionized by radio frequency, microwave frequency, high voltage alternating current or direct current under the room temperature (20 °C-40°C) and atmospheric pressure (*p* ≈ 1 atm, Tg ≈300 K). CAP releases a large number of interactive free radicals and excitatory substances in response to an electric field, UV radiation, and heat. CAP has received increasing attention in the field of medicine over the past few years, with demonstrated efficacy in bacterial ablation [218] and wound healing [219]. Its treatment efficacy in resolving complex diseases such as cancers [220] and joint degenerative syndromes [221] has been gaining increasing attention, indicating its dual role in redox regulation and its multifaceted potency in biomedical applications.

CAP takes on its biological effects primarily through modulating cellular redox homeostasis [222, 223]. Regarding its pro-oxidative roles, CAP has been considered capable of elevating the cellular oxidative level to exceed the death threshold of diseased cells. For instance, by imposing cells with additional oxidative stress that selectively push transformed cells to the death state without harming their healthy peers, CAP has been shown capable of resolving a plethora of cancers including, e.g., triple negative breast cancers [224, 225], bladder tumors [226], prostate cancers [227], liver carcinomas [228] and skin cancers [229], as well as sensitizing diseased cells to existing therapeutics such as targeted therapy [230], chemotherapy [231, 232] and immunotherapy [233–235].

Although CAP induces various forms of programmed cell death by imposing oxidative stress on diseased cells when used at relatively high concentrations, it has been shown to activate intracellular anti-oxidant pathways to counteract oxidative stress at low to mild dosages. Several antioxidant signaling pathways have been implicated, with Nrf2 being the most frequently reported. For example, it has been shown that CAP promoted the intracellular anti-oxidant activity by enhancing the expression of Nrf2 and facilitating its nuclear translocation in treating vitiligo [236]; and activated the DNA damage repair machinery in mitigating radiation-induced skin damage by enhancing Nrf2 nuclear translocation [237].

The efficacy of CAP in treating AD has been reported in both pre- and clinical studies. For instance, by treating an animal AD model six times within 17 days, the dermatitis severity score (DSS), TEWL value, mean epidermal thickness, and serum IgE level significantly decreased by the end of the study [238, 239]. A pilot clinical study including 22 patients with mild-to-moderate AD showed that CAP treatment (performed three times at 0, 1, and 2 weeks, and clinical severity indices evaluated at 0, 1, 2, and 4 weeks after treatment) significantly reduced the AD antecubital severity (ADAS) score and decreased the proportion of *S. aureus* in the microbial population of skin lesions [239, 240]. At the molecular level, CAP was found capable of deactivating NF-κB, decreasing the expression of cytokines or chemokines such as IL-12 and CCL17, and increasing the level of activated Tregs through elevating cellular ROS levels [239] (Fig. 3).

Despite the promising potential of CAP in dermatology, several limitations, including biological, technical, and clinical aspects, hinder its clinical translation. The first challenge involves biological and mechanistic limitations. The therapeutic efficacy of CAP is primarily limited to superficial skin lesions owing to its poor penetration into deeper tissue layers. Although microneedle-assisted delivery systems show promise in enhancing transdermal depth, further optimization is necessary [239, 241–243]. However, the mechanism of action of CAP remains unclear. Although its effects are mediated by complex reactive oxygen and nitrogen species (RONS), the precise molecular pathways involved are not fully understood, hindering the development of rationally designed therapies [239, 241]. Moreover, current research lacks comprehensive disease-specific data. Most studies have been conducted in vitro, with only a limited number of animal models or clinical trials available. This scarcity of detailed signal transduction analyses and condition-specific mechanistic studies restricts the advancement of targeted and personalized treatment approaches [238, 239]. The second major challenge involves technical and standardization issues. A critical limitation is the lack of unified protocols: currently, there lack of standardized guidelines for CAP device parameters, treatment frequency, or duration, resulting in significant variability in both reproducibility and therapeutic outcomes [239, 244]. Furthermore, substantial challenges exist in dosage quantification because accurately measuring CAP dosage and intensity remains technically difficult. This limitation complicates both treatment optimization and clinical outcome prediction [239, 244]. The third category pertains to clinical and safety concerns. However, there is limited evidence supporting the safe and effective clinical application of CAP. Thus, well-designed randomized controlled trials are urgently needed to establish optimal treatment regimens and adapt them to various dermatological conditions, as well as to carefully balance therapeutic efficacy to achieve patient comfort and treatment adherence. Moreover, its long-term safety profile remains uncertain, particularly regarding the effects of repeated CAP exposure on the skin microbiome and systemic immune function, a knowledge gap that warrants comprehensive longitudinal studies. Additionally, significant inter-patient variability in treatment response has been observed, with outcomes influenced by factors such as skin type, medical history, and genetic predisposition, underscoring the critical need for personalized treatment approaches [245]. Although a recent small-scale trial showed clinical improvement in AD syndromes in response to CAP treatment, larger multicenter studies are required to validate its efficacy and safety [240]. Addressing these challenges through interdisciplinary collaboration is crucial for advancing CAP toward clinical adoption.

In addition to CAP, magnetic nanoparticles, capable of increasing cellular redox levels under the influence of applied constant magnetic fields (CMFs) and electromagnetic fields (EMFs), can also be used to treat chronic AD [246]. For instance, maghemite (γ-Fe_2_O_3_) nanoparticles reduce ROS and exert an effective therapeutic effect on AD, taking advantage of the magneto-mechanochemical effect [247].

## Conclusion

AD, a complex dermatological condition with a high disease burden, lacks an effective cure and has minimal side effects. By comprehensively reviewing the disease diagnosis, pathogenesis, manifestations, and risk factors, we emphasized the criticality of redox homeostasis in managing the onset and disease severity of AD and, importantly, proposed a ‘Wound Healing Model’ to distinguish the differential roles that redox homeostasis plays during AD progression in the acute and chronic phases. While the acute phase is characterized by an overtly high ROS level that renders this stage pro-inflammatory, the chronic phase is characterized by insufficient redox levels that cannot effectively trigger the death of overproliferative cells. Based on this model, we categorized current topical and systemic therapeutics for AD management based on their regulatory effects on cellular redox levels. Specifically, approaches for treating acute AD include antioxidants, probiotics, molecular hydrogen, ROS-scavenging nano-materials, pro-oxidative therapeutics, such as blue light irradiation, and the topical use of HClO or its derivatives. As a critical insight, we introduce the unique benefits of CAP in treating AD, especially chronic AD. While acute AD can be treated with multiple antioxidative approaches, including low-to-mild doses of CAP, chronic AD is more problematic and is characterized by abnormal epidermal thickening. CAP may be innovatively applied to rewire the progression of this disease phase back to the acute stage, or to directly induce the death of pathogenic cells, followed by the use of canonical modalities for acute AD treatment. Our insights may not only conceptually advance our understanding of the pathological progression of AD but also shed light on innovative strategies for managing AD, especially chronic AD, which is currently not cured.

Although CAP is promising for use in AD management, it is challenging to precisely control its therapeutic outcome because the effect of CAP on cells is highly dependent on the concentration and type of free radicals it contains. This requires an appropriate definition of the dosage of CAP that can accurately reflect its cellular effects, characterization of biomarkers for dosage evaluation, and establishment of technical facilities capable of dynamically monitoring and tuning the CAP dosage. With the rapid development of other fields, especially artificial intelligence (AI), it is possible to automatically calculate cellular ROS levels based on predefined redox-sensing indexes and determine the dosage tuning direction and amount to achieve the desired therapeutic outcome. Additionally, the limited penetration depth (approximately 2 mm) of direct CAP ejection largely restrains its clinical efficacy. Resolving this technical obstacle through delicate equipment design is an important research topic. Because CAP can be applied in the form of a liquid or hydrogel by exciting these materials, microneedles can be deployed to facilitate the transdermal delivery of CAP-activated liquids. In addition, with the advancement of nanotechnologies, the use of nanomaterials such as exosomes to enhance the delivery efficacy of CAP-activated materials is promising, and synergizing CAP with materials such as MOF may further enhance the treatment efficacy with acceptable side effects. These are promising areas that require intensive exploration.

We underpin the essence of maintaining the integrity of redox homeostasis for treating AD in different phases and propose the unique benefits of CAP in curing this disease. It is worth mentioning that redox-modulating therapies may benefit from a combination with immunotherapies, which is another critical portfolio of AD therapeutic modalities. Redox-modulating therapies exploit the distorted redox balance of pathogenic cells, but face challenges with precise dosing control for desirable outcomes and perhaps limited efficacy as monotherapy. Immunomodulating therapies harness the immune system to directly deliver rapid and potent efficacy but carry risks of immunosuppression-related adverse events, high costs, and variable response durability, necessitating ongoing treatment. Thus, redox modulators and immunomodulators may be considered as adjunctive options and primary agents for AD treatment, respectively, to enhance therapeutic efficacy and reduce adverse effects.

The insights from this study can be extended beyond AD and CAP. Almost all pathological syndromes, including acute and chronic AD, are rooted in disturbed redox homeostasis, which imposes either oxidative or reductive stress on cells. Therapies such as magnetic fields, high-voltage electric pulses, phototherapy, ultrasound therapy, and thermal therapy have similar mechanisms of action once they are appropriately used. These naturally existing powers, including CAP (the fourth state of matter), may convey significant medical therapeutic potential with acceptable or minimal adverse profiles, far beyond what artificial agents can achieve.

Several aspects require further validation. First, the clinical efficacy of CAP in the treatment of human AD remains to be validated through well-designed large-scale clinical trials. Although these preliminary findings are promising, more robust clinical evidence is necessary to confirm its therapeutic potential in chronic AD. Second, although the oxidative activity of ROS appears to be lower in chronic AD than in the acute phase, this conclusion was not directly supported by the redox measurements of skin biopsy samples. Future studies incorporating the redox profiling of lesional skin tissues at different AD stages are essential to validate and refine our mechanistic insights.

## Data Availability

Not applicable.

## Abbreviations

AD: Atopic dermatitis
ADAS: AD antecubital severity
AhR: Aryl hydrocarbon receptor
AI: Artificial intelligence
AMP: Antimicrobial peptides
ARNT: Aryl hydrocarbon receptor nuclear translocators
APCs: Antigen-presenting cells
ASC: Apoptosis-associated speck-like protein containing a CARD
BAX: Bcl-2-associated X protein
CAP: Cold atmospheric plasma
cAMP: Cyclic adenosine monophosphate
CAT: Catalase
CCL: Chemokine (C–C motif) ligand
CFS: Cell-free culture supernatant
CGRP: Calcitonin gene-related peptide
CMFs: Constant magnetic fields
COX: Cyclooxygenase
DAOSD: Dupilumab-associated ocular surface disease
DCs: Dendritic cells
DHA: Docosahexaenoic acid
DHFR: Dihydrofolate reductase
DLQI: Dermatology life quality index
DNP-MR: Dynamic nuclear polarization magnetic resonance imaging
DPI: Diphenyleneiodonium
DSS: Dermatitis severity score
EASI: Eczema area and severity index
ECP: Eosinophil cationic protein
EDN: Eosinophil-derived neurotoxin
EMFs: Electromagnetic fields
EPA: Eicosapentaenoic acid
8-OHdG: 8-Hydroxy-2’-deoxyguanosine
FAK: Focal adhesion kinase
FKBP12: FK506-binding protein 12
FLG: Filaggrin
GSH: Glutathione
GPAT: Glutamine phosphoribosyl amidotransferase
GPT: Glutamic-pyruvic transaminase
GPX: Glutathione peroxidase
GR: Glutathione reductase
GR: Glucocorticoid receptor
GST: Glutathione-s-transferase
HBOT: Hyperbaric oxygen therapy
HClO: Hypochlorous acid
HDAC: Histone deacetylase
H_2_O_2_: Hydrogen peroxide
HNE: 4-Hydroxy-2-nonenal
HW: Hydrogen water
IDH3A: Isocitrate dehydrogenase 3 [NAD] subunit alpha
IFN: Interferon
IgE: Immunoglobulin E
IGA: Investigator's global assessment
IL: Interleukin
ILCs: Intrinsic lymphocytes
IMPDH: Inosine monophosphate dehydrogenase
ISAAC: International study of asthma and allergies in childhood
IL-4Rα: IL-4 receptor alpha
JAK: Janus kinase
KLK5: Kallikrein 5
LCs: Langerhans cells
LEKT1: Lymphoepithelial Kazal-type-related inhibitor 1
MAPK: Mitogen-activated protein kinase
MAG: Magnoflorine
MDA: Malondialdehyde
MOF: Metal organic framework
MPA: Mycophenolic acid
MRPS6: Mitochondrial ribosomal protein S6
NaClO: Sodium hypochlorite
NGF: Nerve growth factor
NF-κB: Nuclear factor kappa-B
NFAT: Nuclear factor of activated T cells
NHEKs: Normal human epidermal keratinocytes
NMF: Natural moisturizing factor
NMN: Nicotinamide mononucleotide
Nrf2: Nuclear factor erythroid 2-related factor 2
N-3 LCPUFA: Omega-3 long-chain polyunsaturated fatty acids
OXPHOS: Oxidative phosphorylation
O_3_: Ozone
PARC: Activation-regulated chemokine
PAR2: Proteinase-activated receptor 2
PDE4: Phosphodiesterase 4
PGF2α: Prostaglandin F2 alpha
PGE2: Prostaglandin E2
PGD2: Prostaglandin D2
PI3K: Phosphoinositide 3-kinase
PKA: Protein kinase A
PTP: Protein tyrosine phosphatase
PMN: Peiminine
PON: Paraoxonase
PO-SCORAD: Patient-oriented scoring AD index
PW: Purified water
ROS: Reactive oxygen species
SCORAD: Scoring AD
SOD: Superoxide dismutase
SPINK5: Serine peptidase inhibitor Kazal type 5
STAT: Signal transducer and activator of transcription
STS: Sodium thiosulfate
ST2: Suppression of tumorigenicity 2
TEWL: Trans-epidermal water loss
Th: T helper cell
TIMP: Tissue inhibitor of metalloproteinases
Tmem79: Transmembrane protein 79
TRPV1: Transient receptor potential vanilloid 1
Trx: Thioredoxin
TSLP: Thymic stromal lymphopoietin
TR: Tryptanthrin
UI: Uncertainty interval
UV: Ultraviolet
UA: Ursolic acid
VEGF: Vascular endothelial growth factor
6-MP: 6-Mercaptopurine
